# Lighting up Polynitrodopamine: from Electropolymerization to Photopatterning of Ultrathin Films

**DOI:** 10.1021/acsami.6c00129

**Published:** 2026-04-08

**Authors:** Marcel Boecker, Julia Moser, Tommaso Marchesi D’Alvise, Christof Neumann, Sean Harvey, Andrey Turchanin, Christopher V. Synatschke, Tanja Weil

**Affiliations:** † Department for Synthesis of Macromolecules, 28308 Max Planck Institute for Polymer Research, 55128 Mainz, Germany; ‡ Institute of Physical Chemistry, 9378 Friedrich Schiller University Jena, 07743 Jena, Germany; § Abbe Center of Photonics (ACP), 07745 Jena, Germany

**Keywords:** nitrodopamine, polynitrodopamine, polydopamine, photocleavage, nanofilms, photopatterning

## Abstract

Polydopamine (PDA) and its derivatives, including polynitrodopamine (PNDA), have attracted considerable attention as versatile materials for surface functionalization and coating. The range of functions in PDA materials increases as new functional groups are conjugated to the dopamine monomer or as copolymers with dopamine derivatives are developed. We report the electropolymerization and photopatterning of PNDA nanofilms as well as free-standing copolymer nanofilms. The insertion of a nitro group into the dopamine structure provides enhanced film properties, including increased film density and photodegradability, thereby facilitating precise nanofilm patterning upon exposure to light. In contrast to PDA, the electropolymerization of PNDA offers to precisely control nanofilm thickness, with film thickness increasing in successive cycles from 19 to 50 nm. While the transfer of pure PNDA films to other surfaces proved challenging, the copolymerization of nitrodopamine and dopamine affords ultrathin films that could be transferred, which gives access to free-standing nanofilms. These 2D PDNA/PDA-based materials reveal photosensitivity as demonstrated through photopatterning yielding smart, customizable surfaces. These results underline the potential of PNDA in advancing surface patterning technologies, with implications for applications in biomedical devices, microfluidic, and sensors.

## Graphical abstract

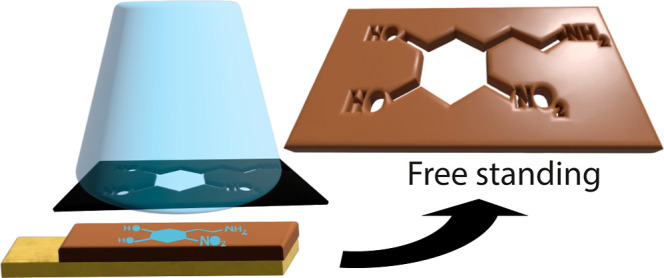

## 1 Introduction

Polydopamine (PDA) and its derivatives have emerged as highly versatile materials for surface functionalization and coating applications.^1^ Since their introduction by Messersmith et al. in 2007, PDA has become an invaluable tool for rapid and efficient surface modification.^2^ Owing to its remarkable versatility, simplicity, and wide-ranging application potential, PDA has been extensively utilized in diverse fields, including drug delivery,^3,4^ photothermal therapy,^4,5^ energy storage devices^6^ such as batteries and supercapacitors, sensors,^7,8^ and filtration systems.^9^ The unique ability of PDA to form coatings in aqueous media at room temperature through a single functionalization step and on virtually any surface^1^ distinguishes it from many other coating materials.

To enhance the functional properties of PDA coatings, considerable research has focused on exploring dopamine analogues.^10^ One particularly promising derivative is nitrodopamine (NDA), typically synthesized by nitration of dopamine.^11^ The incorporation of the nitro group into the dopamine structure significantly alters its chemical properties: the electron-withdrawing nature of the nitro group lowers the p*K*_a_ of the catechol hydroxy groups, thereby increasing their acidity and hydrogen bonding capacity, and improving their oxidation stability.^12^ Moreover, the nitro group enhances the reactivity of NDA toward nucleophiles,^13^ thereby increasing its affinity for coordination with metal oxides.^14^ Additionally, the introduction of the nitro group imparts photosensitive properties to NDA, enabling light-triggered bond cleavage.^15–18^ This photocleavable property has been exploited in various applications, including the development of smart surfaces that can be selectively modified or released upon light exposure.^19,20^

Previously, NDA has been utilized in the creation of photoresponsive hydrogels.^19^ For instance, poly­(ethylene glycol) (PEG)-based hydrogels incorporating NDA as cross-linkers exhibit robust and dynamic underwater bonds that can be specifically cleaved upon light exposure, enabling controlled hydrogel degradation in aqueous environments.^19^ In another example, NDA has been employed for the end-functionalization of polymers such as PEG, allowing the formation of covalently cross-linked gels under oxidative conditions or through metal-mediated cross-linking using Fe^3+^ ions.^21^ By applying masks during light exposure, it is possible to create patterned hydrogels where cross-links are selectively removed from irradiated areas while remaining intact in unexposed regions, thus providing precise spatial control (with patterning 200 μm wide) over the hydrogel properties.^19^ Furthermore, NDA can form photocleavable and biocompatible coatings on solid surfaces when immersed in an alkaline NDA solution, resulting in homogeneous and transparent layers.^19^

For the preparation of ultrathin PDA films, electropolymerization has emerged as a highly effective technique that allows precise control over coating thickness, rapid deposition rates, and the formation of uniform nanofilms on conductive surfaces.^22^ During this process, dopamine derivatives are oxidized at an electrode, initiating polymerization and cross-linking,^23^ which can be tracked via cyclic voltammetry.^24^ Such films have been used in ion sieving^9^ and soft actuators.^25,26^ However, nanostructuring of ultrathin films is essential for various applications, as it significantly enhances film properties by introducing controlled nanoscale features and enabling precise molecular interactions.^27,28^ This results in improved performance for biosensing,^29^ optoelectronics,^30^ and smart coatings.^31^ Previous strategies include DNA-enzyme patterning on DNA origami (<100 nm),^32,33^ photoinitiator-based methods (∼0.8 μm resolution),^34^ and UV-triggered polymerization under photomasks (0.5 mm × 0.5 mm),^35^ though these approaches often face limitations such as cytotoxic residues or nonspecific background polymerization. Patterned electrodes have also enabled spatial control (4 μm × 21 μm),^36^ but lack scalability for complex geometries. Therefore, achieving high-resolution PDA microstructures over larger areas remains challenging due to the material’s inherent chemical stability and cross-linked nature.

Here, we report the first synthesis and photopatterning of electropolymerized PNDA-based nanofilms. PNDA uniquely integrates the robust adhesion properties of PDA, the photosensitivity of NDA, and the precise nanofilm formation afforded by electropolymerization. The photopatterning of PNDA ultrathin nanofilms or PNDA/PDA free-standing nanofilms enables straightforward customization of functional surfaces with high spatial resolution using a simple light source. This approach establishes a versatile platform for various applications such as phototriggered drug release, bioactive surface modification, and biosensor regeneration.

## 2 Materials and Methods

### 2.1 Materials

Gold-coated (1000 Å) microscope slides (Sigma-Aldrich) were cut using a diamond tip. Phosphate buffer (pH 7, 100 mM) was prepared using sodium phosphate dibasic anhydrous (99%) and sodium phosphate monobasic (99%) (Sigma-Aldrich) in Milli-Q water. Carbonate buffer (pH 10, 100 mM) was prepared using sodium bicarbonate (>99.7%) and sodium carbonate (>99.8%) from Sigma-Aldrich in Milli-Q water. A 10 w % Poly­(vinyl alcohol) (PVA) solution was prepared by dissolving PVA (9.5–10.5 kDa, 80% hydrolyzed, Sigma-Aldrich) in Milli-Q water. Gold-coated quartz crystals (6 MHz) (Metrohm) were used for electrochemical quartz crystal microbalance measurements. All other chemicals were purchased from Sigma-Aldrich.

### 2.2 Nitrodopamine Synthesis

The synthesis was carried out using a literature-known method.^19^ First, dopamine hydrochloride (0.5 g, 2.6 mmol, 1 equiv) and NaNO_2_ (0.63 g, 9.1 mmol, 3.5 equiv) were dissolved in 15 mL Milli-Q water. Twenty % sulfuric acid (2.5 mL) was added to the cooled solution (0 °C) under vigorous stirring. A yellow solid immediately precipitated. It was collected by suction filtration and washed several times with cold water and cold methanol. Afterward, the solid was dried under vacuum and rotary evaporation. ^1^H NMR (300 MHz, DMSO-*d*_6_): δ (ppm): 3.05 (4H, s), 6.55 (1H, s), 7.48 (1H, s). The NMR spectra was measured with a Bruker Spectrospin (300 MHz).

### 2.3 ^1^H NMR

The quantitative proton NMR spectra were recorded with the Bruker AVANCE NEO 700 system. The spectra were obtained with π/2 pulse lengths of 14.0 μs and 8 scans. The relaxation delay was 100 s, also to measure the reference signal dimethylsulfone (DMS) and DMSO quantitatively at 298 K. All spectra were internally referenced to the residual proton signals of the deuterated solvent (DMSO-D5H).

### 2.4 Electropolymerization of PNDA

The electropolymerization was done by cyclic voltammetry using a Metrohm Autolab N series potentiostat (AUTOLAB PGSTAT 204) with a standard three-electrode configuration. A gold-covered microscope slide was used as the working electrode (WE) with typical sizes of 2.5 cm by 1.5 cm, Ag/AgCl (3 M KCl) as the reference electrode, and a gold wire as the counter electrode. The WE was pretreated with Ar plasma for 10 min at two mbar pressure, using an Expanded Plasma Cleaner (PDC-001 (115 V) | PDC-002 (230 V), from Harrick Plasma) to remove any organic impurities. The nitrodopamine (1 mg/mL) was dissolved in DMSO (2.5% of the final volume) and added to a phosphate buffer (0.1 M, pH 7), which was flushed with Nitrogen gas for 15 min before the experiment. The reaction solution was poured into a 35 mL electrochemical cell (Metrohm), and the electrodes were immersed in the solution. All but the top 0.7 cm of each electrode was submerged, allowing connection to the potentiostat. A potential was applied and cycled from 1.2 V to −0.5 V, with a scan rate of 0.01 V/s.

### 2.5 Electropolymerization of PNDA/PDA

The electropolymerization was done by cyclic voltammetry using a Metrohm Autolab N series potentiostat (AUTOLAB PGSTAT 204) with a standard three-electrode configuration. A gold-covered microscope slide was used as the working electrode (WE), Ag/AgCl (3 M KCl) as the reference electrode, and a gold wire as the counter electrode. The WE was pretreated with Ar plasma for 10 min at 2 mbar pressure to remove any organic impurities. Dopamine (0.5 mg/mL, 1 equiv) was dissolved in phosphate buffer (0.1 M, pH 7), which was flushed with Nitrogen gas for 15 min before the experiment. Nitrodopamine (1 equiv) was dissolved in DMSO (2.5% of the final volume) and added to the dopamine solution. The electrodes were immersed in the dopamine/nitrodopamine solution, and a potential was applied and cycled from 1.2 V to −0.5 V (starting at 0.5 V), with a scan rate of 0.01 V/s.

### 2.6 eQCM Characterization

An electrochemical quartz crystal microbalance measurement was conducted in a 3 mL electrochemical cell using an Ag/AgCl reference electrode and gold counter electrode by minimizing the driving force prior to starting the measurement.

### 2.7 Fourier Transform Infrared Spectroscopy

The infrared spectra of the film on gold were obtained by grazing-angle reflectance FTIR (Vertex 70, Bruker) after purging the sample with dry air for 15 min and recording four spectra at 3000 scans with an interval of 1 min between each one.

### 2.8 X-ray Photoelectron Spectroscopy

X-ray photoelectron spectroscopy (XPS) was measured using a K-Alpha X-ray Photoelectron Spectrometer System (Thermo Fisher Scientific) with a monochromatic X-ray source (Al K_α_) with a spot diameter of 400 μm and an electron detector with an energy resolution of 0.5 eV. The spectra were calibrated using the C 1s peak (284.6 eV) and fitted using Voigt functions after background subtraction. In order to remove the topmost layer, Ar^+^-ion sputtering was performed using the ion gun of the XPS system with an energy of 500 eV for 15 s.

### 2.9 Atomic Force Microscopy

Atomic force microscopy (AFM) was employed to physically characterize the film, measuring the morphology, surface roughness, and thickness of the polymer films on the gold electrode. This was achieved by scratching the films with a plastic tip immediately after preparation. The scratch profile was then recorded using an AFM (Park NX20) equipped with a cantilever that has a resonance frequency of 70 kHz and an elastic constant of 2 N/m.

### 2.10 Transferring the Film

The film was incubated in a carbonate buffer (0.1 M, pH 10) for 30 min to enhance further cross-linking. Subsequently, cyclic voltammetry was carried out to weaken the interaction between the film and the gold electrode. A potential was then applied and cycled (three times) from 1.2 V to −0.8 V at a scan rate of 0.1 V/s. The film was then washed with Milli-Q water to remove any remaining buffer residues and dried under a nitrogen flow. A sacrificial PVA layer was applied by drop-casting a 10 w % PVA solution onto the film and drying it at 40 °C for 30 min. Afterward, the film was mechanically lifted from the electrode due to the PVA sacrificial layer and transferred onto a new substrate of choice by pressing the film on. Finally, the PVA layer was dissolved by immersing the sample in water for 1 h.

### 2.11 Photodegradation and FITC Release

The films were prepared according to the protocol described above, except that a transparent gold electrode was employed as the working electrode. Then, they were incubated with a FITC solution (1 mg/mL in phosphate buffer (0.1 M, pH 8.5) with 5% DMSO) for 24 h at room temperature. Afterward, they were rinsed with Milli-Q water and incubated in phosphate buffer (0.1 M, pH 7) for 3 days to solubilize any FITC not covalently bound. The films were then rinsed again with Milli-Q water and dried under a nitrogen flow. For the release of FITC, the films were placed in a cuvette containing phosphate buffer (0.1 M, pH 7) and irradiated with a 365 nm LED for 2 h from behind (through the gold electrode). A fluorescence spectrum was measured every minute while the UV LED was blocked with a shutter.

### 2.12 Photopatterning and Degradation Kinetics

The film was placed in a Petri dish and covered with a photomask. Three microscope slides were used as spacers. Then, a solution of phosphate buffer (0.1 M, pH 7) with ascorbic acid (1 M) was added until it covered the entire film under the photomask. A 365 nm LED (ThorLabs) was positioned 5 cm above the photomask, and the film was irradiated for 3 h at 14.56 mW/cm^2^. Afterward, the film was rinsed with Milli-Q water to remove any remaining buffer residues and then dried under nitrogen flow.

For the degradation kinetics, the same irradiation setup was used, just that instead of placing a photomask on top of the film, approximately half of the film was covered with aluminum foil. The Films were irradiated for 30, 60, and 120 min, respectively.

### 2.13 Electropolymerization of PDA

For the electropolymerization of PDA within the patterned PNDA, the previously described three-electrode setup was used. In this configuration, a 10 cycle patterned PNDA film on the gold substrate was used as the working electrode. A dopamine solution (1 mg/mL) was prepared in a phosphate buffer (0.1 M, pH 7), which had been bubbled with nitrogen for at least 15 min prior to experimentation. The electrodes were then immersed in the dopamine solution, and the potential was swept between 0.5 V and −0.5 V for 5 cycles, at a scan rate of 0.01 V/s. Following the polymerization, the film was thoroughly washed with Milli-Q water and dried under a flow of nitrogen.

### 2.14 Profilometry

The film thickness and roughness were measured using a KLA-Tencor Stylus Profiler model P7 (KLA Corporation, California, USA). A scratch was made in the polymer film using a plastic tip to determine the thickness. For thickness determination of the pattern, a section of the pattern was chosen for the profilometer to measure the entire pattern and part of the remaining film on both sides. For the scratch, a length of approximately 300 μm, and for the pattern, up to 900 μm was analyzed. The measurements were taken with the following parameters: scan speed 10 μm/s, sampling rate 100 Hz, applied force 0.50 mg, vertical range 65 μm, and vertical resolution 0.0391 Å.

## 3 Results and Discussion

### 3.1 Electropolymerization of PNDA Films

The NDA monomer was synthesized following a previously established protocol,^19^ and its successful synthesis was confirmed by ^1^H NMR (Figure S1). To fabricate PNDA films, the NDA monomer was subjected to electropolymerization using cyclic voltammetry (CV) as illustrated in Figure 1a. The electropolymerization process was carried out in a conventional three-electrode system consisting of a gold slide (working electrode), a gold wire (counter electrode), and an Ag/AgCl reference electrode.^10,37^ Although NDA shares structural similarities to dopamine, which can undergo electropolymerization with an upper vortex potential of +0.5 V and a lower vortex potential of −0.5 V,^10^ these potential settings, optimized for dopamine, did not support robust film formation of PNDA on the electrode surface. Therefore, the electropolymerization conditions were modified to include an upper vortex potential of +1.2 V and a lower vortex potential of −0.5 V to ensure adequate oxidation of NDA, facilitating effective nanofilm formation (Figure 1b).

**Figure 1:**
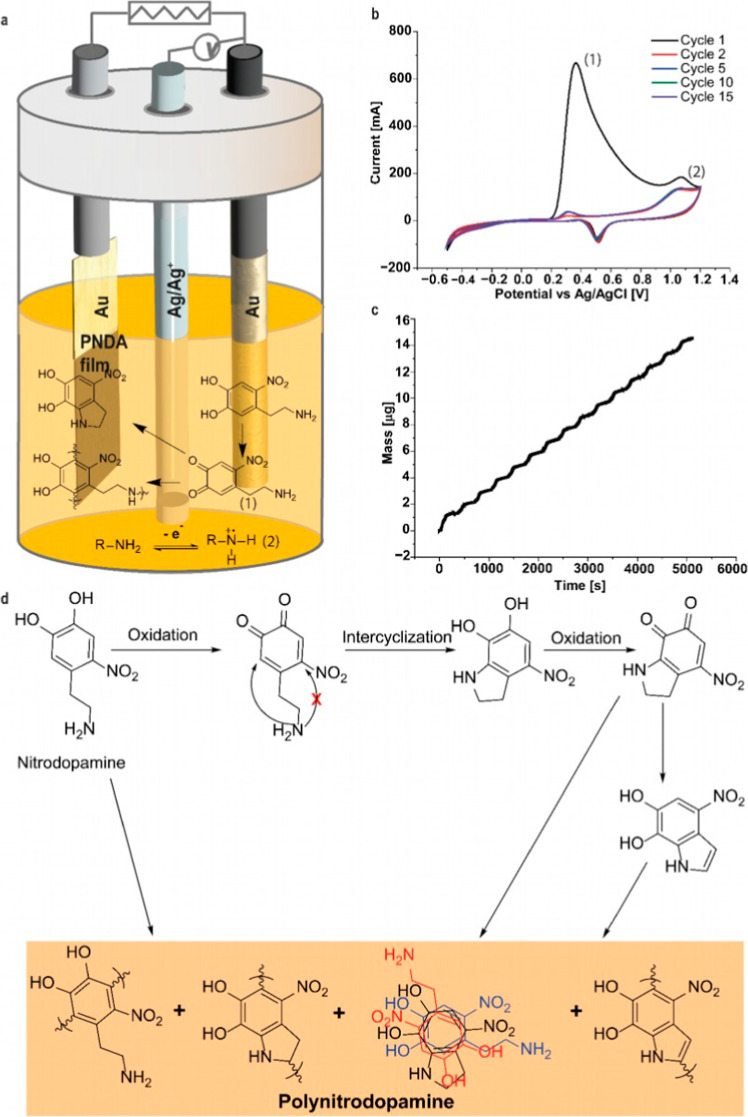
(a) Schematic illustration of the three-electrode electropolymerization setup (adapted with permission from ref 9 copyright 2024 Wiley-VCH.), with the initial oxidation steps of NDA, which lead to film formation on the electrode surface. (b) Cyclic voltammogram of the electro-polymerization of NDA with the peak indication for the respective oxidations. (c) Increase in mass over time of a 15 cycle PNDA film during electropolymerization on an eQCM electrode. (d) Proposed electropolymerization mechanism for the formation of PNDA, based on the mechanism of PDA, with the crossed arrow indicating the major difference between the mechanisms of PDA and PNDA formation.

During electropolymerization, the cyclic voltammogram (CV) revealed a distinct oxidation peak at +0.36 V (Figure 1b), corresponding to the oxidation of the catechol to the quinone group (1, Figure 1a). Additionally, a second oxidation peak was observed at +1.07 V, which is attributed to the oxidation of the amine group at the side chain and that is consistent with the known oxidation potentials of dopamine (1.22 V vs SCE).^38^ After the first cycle, a marked reduction in current was detected, while subsequent cycles exhibited no further decrease. This observation implies that the majority of PNDA deposition likely took place during the initial cycle. However, this seems unlikely, as further mass deposition can be observed in the eQCM (Figure 1c). It is more probable that NDA monomers underwent oxidation during the initial electropolymerization cycle, leading to a limited availability of monomers for the subsequent polymerization cycles. This interpretation aligns with the sharp drop in current after the first cycle, while mass deposition continued at a relatively consistent rate (Figure 1c), as discussed below, suggesting that the restricted diffusion of NDA monomers to the electrode surface limited their availability for oxidation in later cycles. Furthermore, the higher mass increase during the first cycle, compared to subsequent cycles, supports diffusion-limited monomer availability after the initial oxidation event.

### 3.2 Controlled PNDA Nanofilm Deposition

To monitor PNDA deposition during electropolymerization, we employed an electrochemical quartz crystal microbalance (eQCM, Figure 1c). A gold-coated quartz crystal served as the working electrode, and the oscillation frequency was continuously recorded to assess the PNDA deposition. A decrease in crystal frequency was observed during the first cycle, indicating the onset of PNDA film formation. Despite the significant drop in current after the first cycle, film growth continued as confirmed by eQCM measurements, as discussed above. Specifically, 1.31 μg of PNDA were deposited on the electrode after the first cycle (340 s), and the mass deposition further increased to 14.53 μg after 15 cycles (5105 s), indicating continuous mass deposition during the entire electropolymerization process.

The thickness and surface morphology of the deposited films were analyzed by atomic force microscopy (AFM). An AFM image of a representative 5 cycle PNDA film, which was scratched using a plastic tip to assess film thickness, is shown in Figure 2b. The thickness of the PNDA films increased with the number of electropolymerization cycles. After five cycles, the film thickness was 18.5 ± 0.5 nm, whereas after 15 cycles, the thickness reached 48.3 nm (Table 1). A direct comparison with the corresponding mass data (Figure S2) reveals an almost perfectly linear correlation between deposited mass and film thickness, indicating homogeneous film growth during electropolymerization.

**Figure 2:**
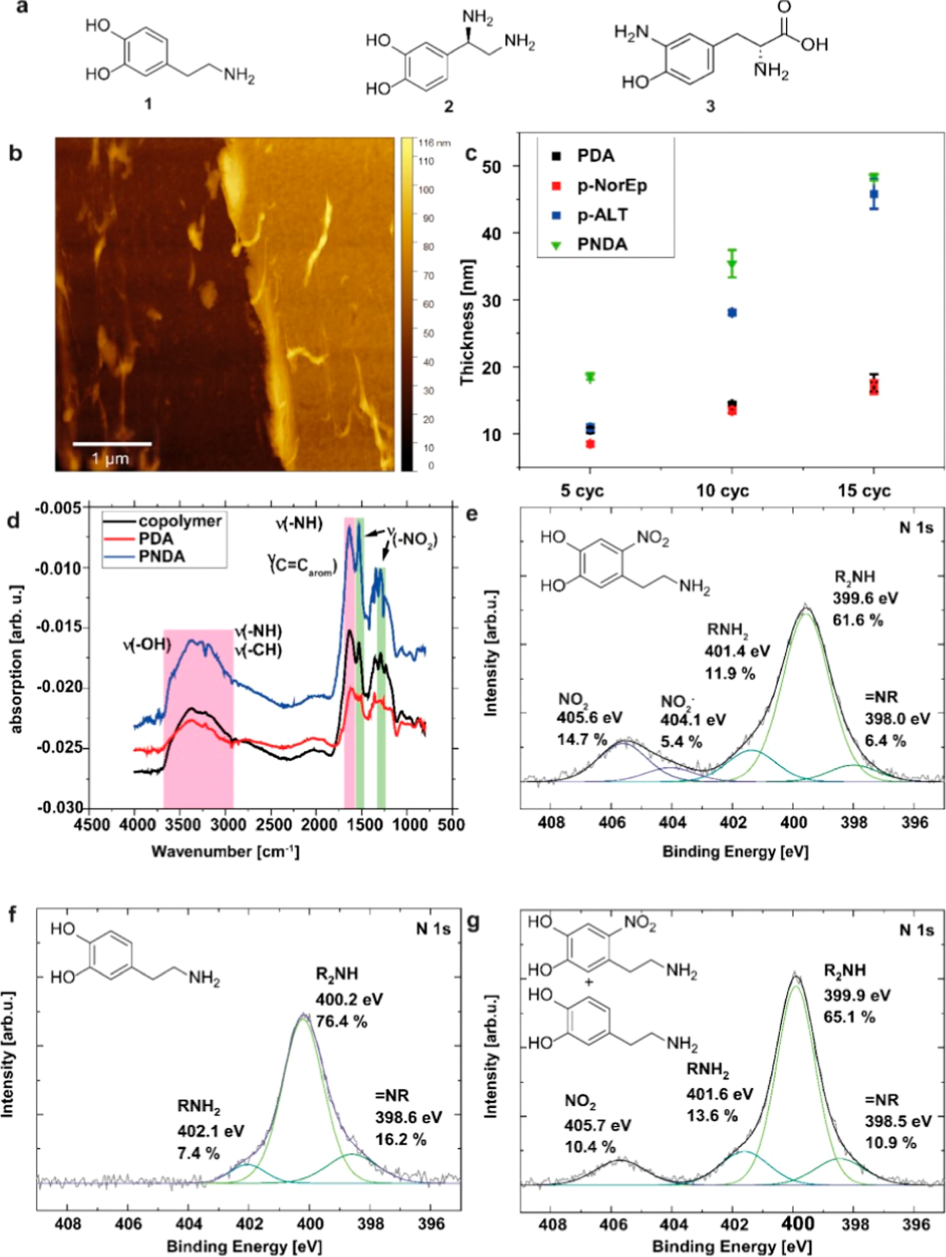
Morphological, spectroscopic, and compositional characterization of PNDA nanofilms compared to related polycatecholamines. (a) Chemical structures of the respective monomers dopamine (PDA), noradrenaline (PNE), and 3,4-dihydroxyphenylalanine (PALT). (b) Atomic force microscopy (AFM) image of the scratched area for a PNDA film (10 cycles). (c) Thickness comparison of PNDA films with literature-known polycatecholamine (PCA) systems for 5, 10, and 15 cycles of electropolymerization determined by AFM scratch assay. Data for PDA, PNE, and PALT were adapted from ref 10. (d) Fourier-transform infrared (FTIR) spectra of PNDA, PDA, and PNDA/dopamine nanofilms, with peaks for the nitro group highlighted in green and other functional groups highlighted in magenta. (e) High-resolution N 1s X-ray photoelectron spectroscopy (XP) spectrum of PNDA nanofilms alongside fitting components. (f) High-resolution N 1s XP spectrum of PDA nanofilms alongside fitting components. (g) High-resolution N 1s XP spectrum of PNDA/PDA nanofilms alongside fitting components.

**Table 1:** Density of PNDA, Calculated from eQCM and AFM Data with the Respective Thickness Determined by AFM in Brackets, and Polydopamine (PDA), Polynorepinephrine (PNE), and poly­(amino-l-tyrosine) (PALT) from Literature^10^

polymer	5 cycles		10 cycles		15 cycles	
	density [g/cm^3^]	thickness [nm]	density [g/cm^3^]	thickness [nm]	density [g/cm^3^]	thickness [nm]
PNDA	2.81	18.5 ± 0.5	2.71	35.4 ± 2.0	3.02	48.3 ± 0.5
PDA	1.79	10.6 ± 0.4	1.91	14.4 ± 0.3	1.75	17.6 ± 1.3
PNE	1.66	8.5 ± 0.3	1.43	13.5 ± 0.4	1.41	17.0 ± 1.0
PALT	1.43	11.0 ± 0.5	1.52	18.9 ± 0.8	1.38	45.8 ± 2.2

The film density was estimated by dividing the mass, which was calculated using the Sauerbrey equation (eq 1) from eQCM measurements, by the volume of the film. The volume was determined by multiplying the electrode area (a circle with a diameter of 1 cm) with the thickness measured by AFM (see Table 1).

1 $$-\Delta f=\Delta m\times C_{f};{\;C}_{f}=0.0815Hz\times {ng}^{-1}\times {cm}^{-2}$$

where Δ*f* represents the frequency change, due to mass deposition, measured via eQCM, m is the mass, also measured by eQCM and C_f_ is the constant of the quartz crystal electrode. Applying this equation allowed us to correlate the mass increase with the observed film thickness, confirming the formation of dense and uniform PNDA films.

The calculated density and the measured thickness of the PNDA nanofilms were listed in Table 1 and they were compared to published data from electropolymerized PDA, polynorepinephrine (PNE), and poly­(amino-l-tyrosine) (PALT) films (Table 1, monomer structures in Figure 2A, comparison Figure 2C).^10^ The thickness of the PNDA films increased from 18.5 nm (5 cycles) to 48.3 nm (15 cycles), which was similar to the thickness of PALT films that increased from 11.0 nm (5 cycles)^10^ to 45.8 nm (15 cycles)^10^ during the electropolymerization cycles. In contrast, PDA and PNE films revealed a reduced film thickness, which aligns with their known self-limiting growth (Figure 2c).^10^ The obtained PNDA films were smooth and had a similar roughness as PDA^10^ after five, ten, and 15 cycles (Table S1).^10^ Interestingly, PNDA films exhibited a significantly higher film density compared to the three reference polymer films, which remained constant independent of the number of electropolymerization cycles. In contrast, the reported densities for PDA, PNE, and PALT films were significantly lower, ranging from 1.38 to 1.75 g/cm^3^ (Table 1). The high PNDA density could be attributed to the incorporation of additional NDA monomers during the electropolymerization process, which could lead to enhanced cross-linking within the film, which would be reflected by the observed higher film density.

To date, with the exception of an NDA dimer isolated from an enzyme-catalyzed oxidation of PNDA, no distinct structural elements of PNDA have been conclusively identified.^39^ To gain deeper insights into the functional groups present in PNDA, we performed Fourier-Transform Infrared Spectroscopy (FTIR). The FTIR spectrum of the PNDA film (Figure 2d) exhibits distinct nitro peaks at 1284 cm^–1^ and 1529 cm^–1^, confirming the presence of nitro groups. These groups are absent in the FTIR spectrum of PDA, while all other characteristic peaks observed for PDA are also present in PNDA.

X-ray Photoelectron Spectroscopy (XPS) of PNDA reveals four distinct nitrogen species (Figure 2e). Three of these species, corresponding to RNH_2_ (401.4 eV), R_2_NH (399.6 eV), and =NR (398.0 eV), are characteristic of PDA and are clearly detectable in the XPS spectrum of PNDA. Furthermore, the spectrum shows clear signals corresponding to the nitro group (404.1 eV, 405.6 eV) with a fraction of ∼20% of the nitrogen content, again confirming its incorporation into the PNDA structure. Accordingly, the detected amount of nitrogen in the PNDA sample (11.2 at %) is significantly higher in comparison to the PDA sample (7.5 at %) (see Table S3 for details). Additionally, the high-resolution XPS spectra of other elements primarily reveal the presence of C–C, C–H, C–O, and C=O bonds, consistent with the structure of PDA and no additional PDNA-specific functionalities were resolved.

Therefore, considering the structural similarity to PDA and the lack of unique spectral features, it is likely that PNDA, similar to PDA, consists of a diverse and complex polymeric network. Due to this similarity, we propose a possible electropolymerization mechanism and a representative structural motif in Figure 1d.

As with PDA, the resulting PNDA polymer is expected to form through a series of oxidative, cyclization, and coupling reactions, yielding a heterogeneous mixture of oligomeric and polymeric species. The presence of the electron-withdrawing nitro group further modulates the reactivity of intermediates, but does not fundamentally change the overall nature of the polymerization process. A precise structural elucidation of PNDA, however, is extremely challenging due to the inherent chemical complexity and lack of uniform repeating units, an issue well-known from PDA research.

### 3.3 Photodegradation and Patterning of PNDA Nanofilms

It has been previously reported that introducing a nitro group to the dopamine structure can lead to the formation of photodegradable materials.^19^ As proof of principle, an electropolymerized PNDA film (10 cycles) was functionalized with FITC through the formation of a thiourea bond (see Figure 3c). This film was irradiated with a 365 nm LED for 2 h, as longer durations resulted in oversaturation of the detector for fluorescent measurements. The degradation of the film was monitored by measuring the fluorescence in the solution (Figure 3a). For comparison, the same procedure was performed with a PDA film. A significant increase in fluorescence intensity was observed only for the PNDA film (Figures 3b and S3), confirming that FITC was released upon light exposure. This demonstrates the potential of PNDA films for light-triggered molecule release, serving as a proof-of-concept for controlled drug delivery.

**Figure 3:**
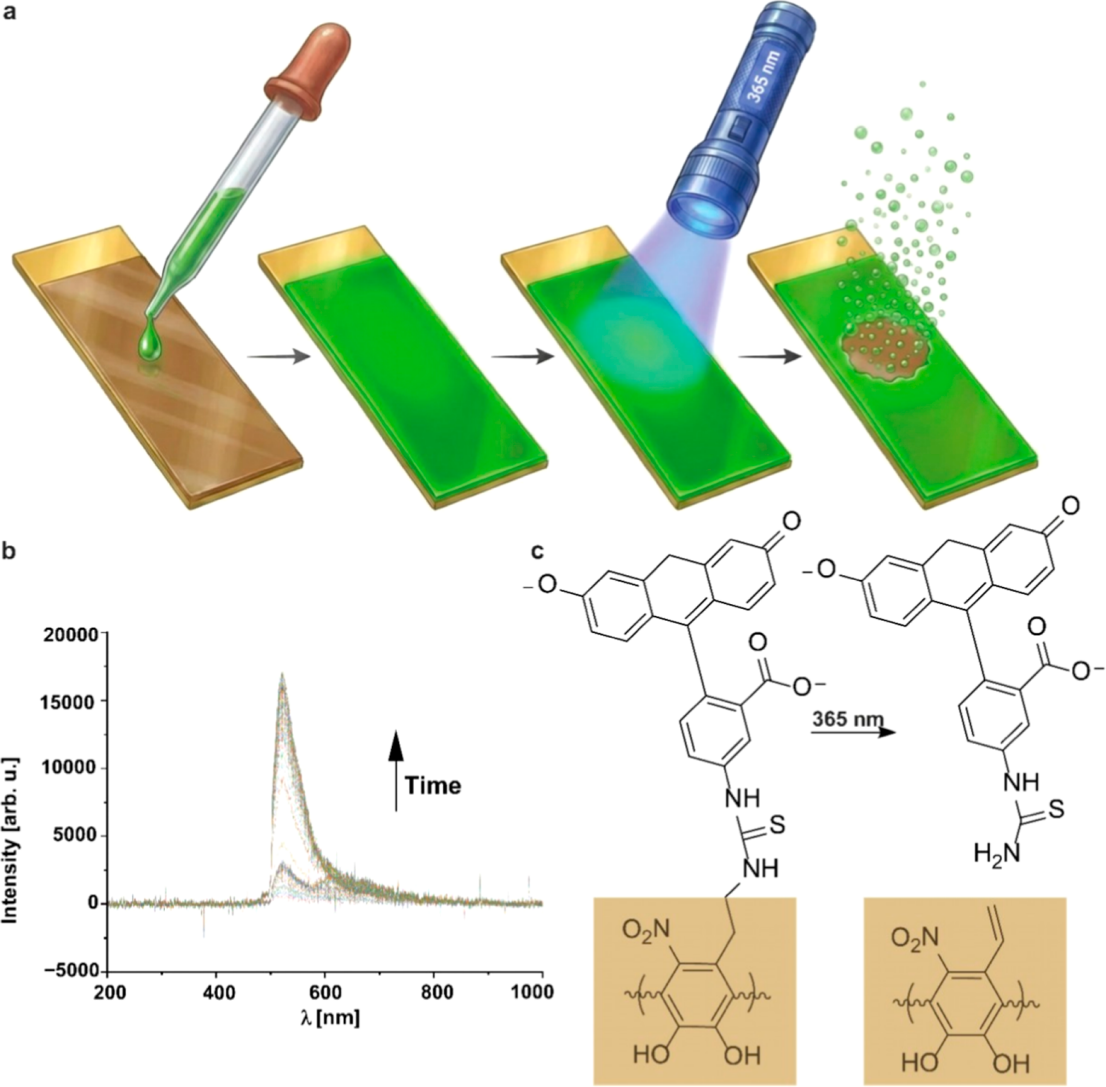
Demonstration of phototriggered functionalization release PNDA using FITC as a model drug. (a) Schematic drawing of the FITC functionalization of a PNDA film (10 cycles) and the respective photodegradation process. The schematic was created with Illustrae using an original image prepared by the authors. (b) The recorded fluorescence spectra were measured over a duration of 2 h during the irradiation of a FITC-functionalized PNDA film with a 365 nm LED. (c) Reaction scheme based on the established structural similarity between PNDA and PDA. The scheme depicts the most probable bond-formation pathway during functionalization via a primary amine, as well as the most likely bond-cleavage pathway during photocleavage, consistent with literature reports on polynitrodopamine.^19^

To further explore the photoresponsive capabilities of PNDA, we investigated whether these properties could also be harnessed for spatially controlled photopatterning. For this purpose, an electropolymerized PNDA film (10 cycles) was covered with a photomask and subsequently exposed to a 365 nm LED light for 3 h (Figure 4a). Initially, when the film was irradiated in its dry state and then rinsed with water, no noticeable patterns appeared. Measurements taken with a profilometer indicated no reduction in thickness (Figure S4), which could either suggest that the photocleavage products remained trapped within the film matrix or that photocleavage did not proceed efficiently in the absence of water. To overcome this limitation, the PNDA film was immersed in phosphate buffer (0.1 M, pH 7) during irradiation in order to detach and remove the photocleavage products. Under these conditions, precise photopatterns with dimensions in the micrometer range (Figure 4b) were obtained after irradiation for 2 h. The patterned film was analyzed using a profilometer to demonstrate removal of PNDA from the irradiated areas (Figure 4c). The step height of the patterned areas was compared to that of a scratch made by a plastic tip on the same film. The measured step height of the photopatterned area was 36 nm, closely matching the 36.2 nm height of the mechanically scratched area, depicted in Figure S5, confirming complete removal of the PNDA film from the irradiated surface areas. The same is true for a 5 and 15 cycle PNDA film, where complete film removal in the patterned areas due to irradiation was also achieved (Figure S6 b and c).

**Figure 4:**
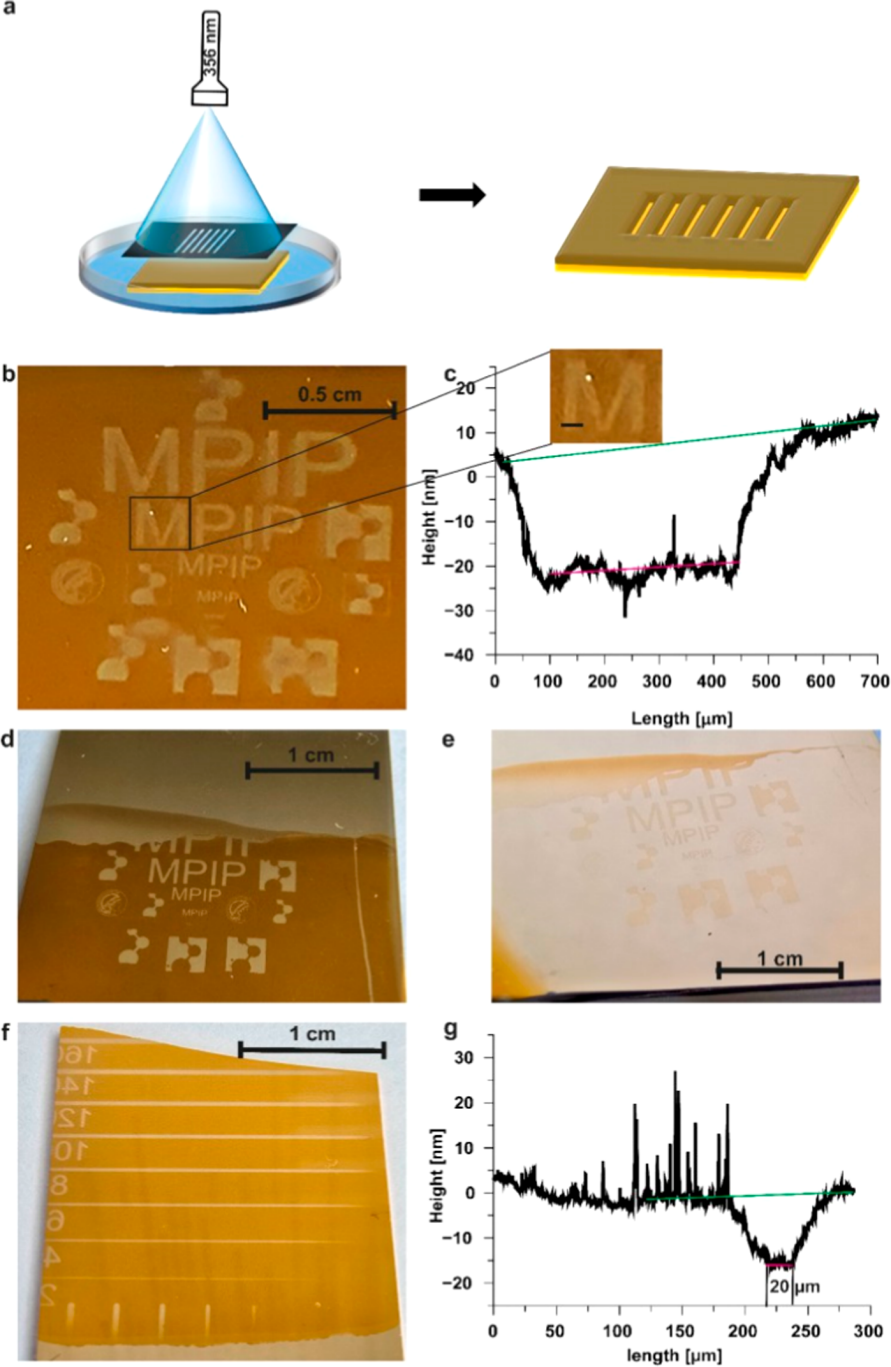
Photopatterning and lithographic characterization of PNDA nanofilms. (a) Schematic illustration of the photopatterning process by placing the film beneath a photomask in phosphate buffer (PB) and irradiating it for 2 h using a 365 nm LED. (b) Optical image of a 10 cycle photopatterned PNDA nanofilm with a pattern width of 1.5 cm. (c) Height profile measured by profilometry of the patterned region with fitting curves for height determination (green: fit of the remaining film; red: bottom of the patterned area), with the measured height profile location indicated in the zoomed-in view of the optical image. (d) Optical image of a 10 cycle photopatterned PNDA nanofilm with a 5 cycle polydopamine (PDA) film deposited inside the pattern. (e) Optical image of the 5 cycle PDA film inside the pattern after selective removal of the surrounding PNDA film. (f) Optical image of a 10 cycle photopatterned PNDA nanofilm with different line widths (20 to 160 μm) to determine the lithography resolution. (g) Corresponding height profile of the 20 μm width line.

In contrast, PDA reference films did not exhibit any pattern formation after irradiation, regardless of whether they were irradiated in the dry or wet state (Figure S6d). This lack of photosensitivity further highlights the unique photodegradable properties of PNDA compared to pure PDA films. These results demonstrate that PNDA films can be precisely photopatterned when irradiated in buffered media, allowing for the controlled removal of degradation products from the irradiated surface areas.

Following these observations, the photodegradation of PNDA films in aqueous buffer can be rationalized by analogy to both PDA’s ability to generate hydroxyl radicals under ultraviolet illumination^40^ and the well-characterized aqueous photochemistry of nitroaromatic substrates.^41^ Therefore, we assume that, under UV irradiation in water, catechol-quinone motifs within PNDA undergo photoexcitation that produce reactive OH radicals, as has been demonstrated for PDA nanoparticles using terephthalic-acid trapping and ESR detection.^40^ These hydroxyl radicals could then attack the nitrophenyl rings of PNDA via an ipso-addition pathway analogous to nitrobenzene photodegradation in aqueous solution (see Figure S7).^41–44^ Here, the nitrophenyl C bearing the nitro group is attacked by the hydroxyl radical, leading to the formation of a hydroxynitrocyclohexadienyl radical. Subsequent fragmentation liberates NO_2_^–^ (and minor NO) and yields aryloxyl radicals, which propagate chain-cleavage and further oxidation on neighboring indole-quinone units. In water, nitrobenzene itself undergoes ultrafast vibrational cooling and primarily returns to its ground state without experiencing direct photolysis.^41^ As a result, the initial degradation of PNDA occurs through hydroxyl radical-mediated attack on the nitroaromatic structure, rather than direct light-induced cleavage of the C–N bond. Finally, facile diffusion of soluble oxidative fragments out of the illuminated regions produces the observed photopatterns in PNDA films, while unmodified PDA films, lacking nitroaromatic sites, remain unetched under identical conditions.

To further validate this hypothesis, a 10 cycle PNDA film was patterned using the method described above. Subsequently, a 5 cycle PDA film was synthesized on the same substrate, allowing PDA to grow exclusively within the patterned regions where the PNDA film no longer blocked the electrode (Figure 4d). The step height within the pattern was measured using a profilometer, while the thickness of the 15 cycle PNDA film was determined by creating a scratch. The difference between the PNDA film thickness and the step height within the pattern corresponded to the thickness of a 5 cycle PDA film (11 nm).^37^ To confirm that only the PNDA film is photodegradable, the entire film was irradiated using the same setup as before, but without the photomask. As a result, the PNDA film was completely removed, leaving behind the PDA film as a negative of the original pattern (Figure 4e), effectively acting as a positive photoresist. Importantly, the thickness of the PDA film remained unchanged after irradiation.

The resolution of the photodegradation process was evaluated by irradiating a 10 cycle PNDA film using a photomask containing line patterns of varying widths (ranging from 20 to 160 μm). The same irradiation setup as described above was used for this experiment. The resulting pattern (Figure 4f) was subsequently characterized using a profilometer. Even the narrowest lines, with a width of just 20 μm, could be clearly resolved and measured (Figure 4g), demonstrating the high spatial resolution achievable with this photopatterning method.

A degradation kinetics study was carried out using 10 cycle PNDA films, which were irradiated for 30, 60, and 120 min. During each irradiation, part of the film was covered to serve as an internal reference. Scratches were made in both the irradiated and protected areas, and the film thicknesses were measured using a profilometer. After 30 min of irradiation, 76% of the original film thickness remained; after 60 min, only 36% was left (Table S2). Following 120 min of exposure, no reliable height measurement could be obtained due to extensive degradation, although small film fragments were still observable on the surface. From these measurements, a degradation rate of 0.0131 min^–1^ was calculated for the PNDA film.

### 3.4 Preparation of PNDA/PDA Copolymer Nanofilms

The transfer of ultrathin films remains challenging due to strong surface adhesion or inadequate structural support, often leading to fragmentation or tearing during handling.^45^ Previously, we developed a method to transfer PDA nanofilms onto various substrates, including nonconductive surfaces.^10^ However, this protocol proved ineffective for transferring PNDA films synthesized on gold electrodes. Despite the structural similarities between PNDA and PDA, the existing transfer method could not detach PNDA films from the gold substrate. This difficulty is attributed to the strong adhesion forces between PNDA and the gold electrode, likely driven by the presence of the nitro group, which enhances electrostatic π–π interactions through π-hole effects.^46^ These interactions may arise from variations in electron density and aromatic orientation, influencing interactions with adjacent π-systems or surfaces.^13,46^ Such π-hole interactions are known to stabilize molecular adhesion on metallic surfaces, as functional groups like nitro groups can modify adsorption modes and strengthen interfacial bonding.^48^

To address these challenges, we developed free-standing PNDA/PDA nanofilms by copolymerizing PNDA and PDA through electropolymerization, combining photosensitivity with reduced adhesion to gold surfaces. A 1:1 monomer ratio of NDA and dopamine was chosen, using the same electropolymerization conditions previously established for pure PNDA. To avoid forming a pure PDA layer on the gold electrode, the cyclic voltammetry process was initiated at 0.5 V. PDA electropolymerization reveals an oxidation peak at 0.3 V,^37^ whereas PNDA exhibits a slightly higher oxidation peak at 0.36 V (Figure 5b), attributed to the electron-withdrawing nitro group, which stabilizes NDA against oxidation. Consequently, dopamine oxidation precedes NDA oxidation, as confirmed by the CV graph (Figure 5b), which revealed an initial oxidation peak derived from both dopamine and NDA (Figure S7) for the first cycle and Figure 5b for cycles two to ten and a broadened peak at 0.28 V, corresponding to the oxidation of catechol units from both monomers (Figure 5b). Subsequent cycles exhibited CV profiles similar to pure PNDA, indicating copolymerization of both monomers.

**Figure 5:**
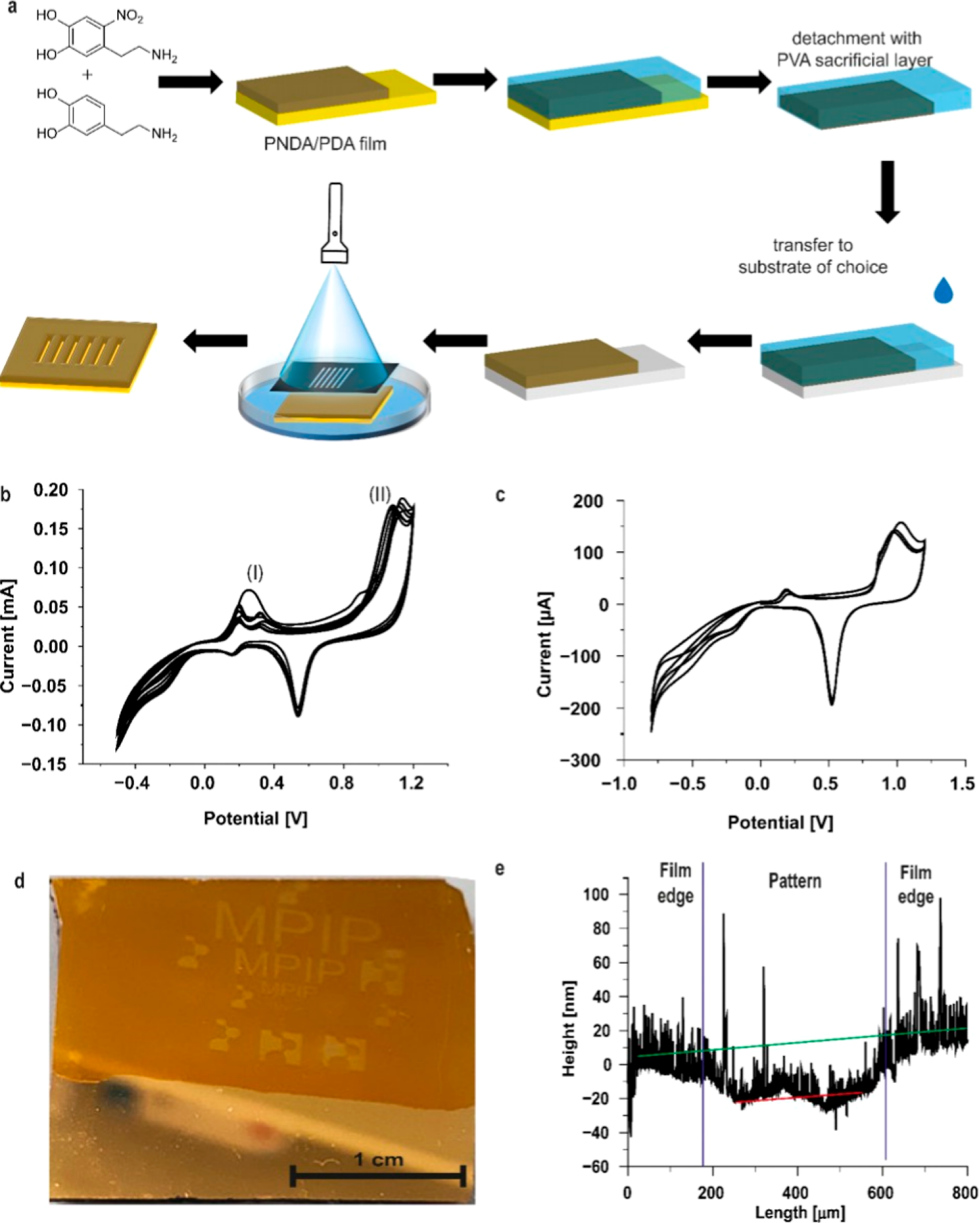
Film removal and photopatterning of PNDA/PDA nanofilms. (a) Illustration of the film removal process with the follow up photoatterning of the PNDA/PDA film. (b) Cyclic voltammogram showing cycles two to ten of the electropolymerization process for the PNDA/PDA nanofilm. It highlights the oxidation peak corresponding to the conversion of catechol to quinone (I). Starting from the third cycle, this peak begins to split, indicating the oxidation of the catechol group in dopamine, followed by the oxidation peak of the catechol group in NDA. Additionally, there is an oxidation peak for the amine group of the side chain (II), which is similar to that observed during the formation of pure PNDA. (c) Cycle voltammogram of the overoxidation to enable removal of the film from the electrode surface. (d) Camera image of the photopatterned PNDA/PDA film with a total width of 2.5 cm and (e) the height profile of the patterning measured with a profilometer with indication of the transition of the film to the patterning (blue lines) and the used fits for the film surface (green) and the inside of the patterning (red) for the height determination.

Incorporating PDA into the PNDA film reduced the overall content of nitro groups from ∼20% of the nitrogen content to ∼10% as shown by XPS (Figure 2g), thereby weakening the film’s adhesion to the electrode. The amount of nitrogen in the sample is 10.4 at % and therefore between the detected values of PDA and PNDA (see Table S3). This modification enabled the successful transfer of free-standing films based on the reported protocol.^37^ Prior to transfer, the film was immersed in carbonate buffer (0.1 M, pH 7) for 30 min to enhance cross-linking. The removal process was conducted using CV in the potential range of −0.8 to 1.2 V for three cycles. The resulting CV graph displayed a characteristic oxidation peak at 1 V and a reduction peak at 0.5 V, indicative of the overoxidation of the PNDA/PDA film and exposure of the bare gold electrode (Figure 5c), consistent with the CV graph of pure PDA.^37^ After overoxidation, a sacrificial poly­(vinyl alcohol) (PVA) layer was introduced to facilitate film transfer. The PVA layer is needed to mechanically support the ultrathin PNDA/PDA nanofilm during the transfer. The intact copolymer film with the PVA layer on top was successfully transferred to a nonconductive glass substrate, as depicted in Figure 4a. The PVA layer was subsequently dissolved by submerging it in water. Profilometer measurements indicated a film thickness of 32.8 nm (Figure S8), which is slightly lower than that of pure PNDA (36 nm). However, the surface roughness increased significantly from 0.4 to 7.8 nm (Table S1), suggesting higher structural inhomogeneity of the copolymer nanofilm.

The presence of the nitro group in the synthesized PNDA/PDA copolymer was confirmed by FTIR analysis, which revealed the characteristic nitro peaks at 1284 cm^–1^ and 1529 cm^–1^ (Figure 2d), similar to those observed in pure PNDA. Additionally, XPS analysis confirmed the presence of increased carbon content and reduced nitrogen content in the copolymer film compared to pure PNDA, attributable to the incorporation of PDA (Table S3 and Figure S13). Despite these changes, the high-resolution spectra displayed the same chemical species as found in pure PNDA (Figure 2g), confirming the successful formation of a copolymer film. These results demonstrate that combining PDA and PNDA effectively reduces film adhesion to the substrate, enabling successful transfer while maintaining the desirable properties of both components. This method provides a practical approach to creating free-standing PNDA/PDA nanofilms for transfer to various substrates.

### 3.5 Photopatterning of Free-Standing PNDA/PDA Nanofilms

After transferring the PNDA/PDA copolymer free-standing films onto another gold slide, we studied their photopatterning properties. Gold slides were selected as receiving substrates due to their smooth surface, which facilitates more accurate height measurements and provides high contrast for pattern visualization. Importantly, the transferred film was subsequently immersed in phosphate buffer and then irradiated with 365 nm UV light for 3 h under a photomask, following the same protocol as described for pure PNDA films (see photopatterning section, Figure 5a). This confirms that the transferred films adhere to the new substrate, even when submerged in solution. After irradiation, the photomask pattern appeared on the nanofilm (Figure 5d). Profilometer measurements of the patterned area revealed a step height of 16.9 nm (Figure 5e), approximately half the thickness of the original PNDA/PDA film. This indicates that either photodegradation was incomplete or that material such as PDA had redeposited during UV irradiation.

We believe that the latter process is more likely. The uneven surface within the patterned region could indicate the formation of a new layer on top of the patterned area. It is known that UV light used for photopatterning could also polymerize dopamine^38^ and therefore, UV-induced radicals generated from dopamine^39^ could have recombined on the patterned surface, resulting in the formation of a new PDA layer. To investigate this hypothesis, we conducted XPS measurements at two locations: one within the patterned area and one from the nonirradiated section protected by the photomask (Figures S9,S10 and S12). After the initial XPS analysis, the sample was subjected to Ar^+^ ion sputtering for 15 s to assess the bulk structure (Figures S10,S11 and S13). For the pure PDA reference film, no changes were observed between the irradiated and nonirradiated regions (Figure S9). Additionally, sputtering did not reveal any differences, apart from a decrease in relative intensity due to reduced material thickness. No visible patterning was observed on the PDA film (Figure S7d). In contrast, the pure PNDA film and the PNDA/PDA copolymer film displayed the nitro group moiety in the masked area (Figures S11 and S13) and before irradiation (Figure 2e,g), but this moiety was absent from the patterned area of the pure PNDA film (Figure S11). Notably, the pure PNDA film showed a higher amount of gold signal from the substrate in the patterned region, indicating that only a thin residual layer remained, likely due to residual fragments on the uneven gold surface.

After 15 s of Ar^+^ sputtering, the pure PNDA film exhibited only the Au signal, confirming the complete removal of the organic film (Figure S12). In contrast, the PNDA/PDA film still displayed organic residues after sputtering (Figure S14), suggesting that the copolymer structure was more resilient to surface degradation. For nonirradiated areas, both the PNDA film (Figure S12) and the PNDA/PDA film (Figure S14) retained material even after sputtering. Interestingly, the nitro group signal was absent in the high-resolution spectra of both films after sputtering, likely due to the interaction between the nitro group and the ion beam. Even though distinct patterns have been achieved after photopatterning of the PNDA/PDA copolymer film, our results indicate that a new PDA layer could form on top of the photopatterns, likely due to radical recombination.

## 4 Conclusion

We introduce PNDA and its copolymer PNDA/PDA as versatile, photodegradable polymeric 2D materials for advanced surface functionalization and photopatterning applications. Electropolymerization of PNDA and PNDA/PDA affords ultrathin nanofilms with tunable thickness in the low nanometer regime. Unlike PDA, PNDA films exhibit film thickness precisely tunable with successive deposition cycles. The PNDA/PDA copolymer network overcomes the strong adhesion of PNDA films to gold electrodes, enabling their transfer to other nonconductive substrates, which broadens their applications and integration into devices.

Moreover, UV-triggered degradation of PDNA and PNDA/PDA films allows the formation of precise microscale patterns (20 μm in width) and the release of embedded molecules, as demonstrated by the release of FITC used as a model compound in a proof-of-principle experiment. The incorporation of the nitro group within the polymer backbone is crucial for enabling this light-responsive behavior, positioning PNDA-based materials as promising candidates for the development of smart, reconfigurable surfaces. The films’ compatibility with phosphate buffer environments, combined with their mechanical robustness and processability, makes them ideally suited for applications in biological environments. Further optimization of the film transfer process and a deeper mechanistic understanding of the photodegradation pathway will be essential to fully exploit the potential of these bioinspired materials. The results of this study highlight PNDA and PNDA/PDA films as innovative photoresponsive coatings and free-standing nanofilms. Our work suggests that PNDA nanofilms and their copolymers hold great potential for applications, particularly as positive photoresists and drug delivery systems.

## Supplementary Material



## Notes

^1^H NMR of nitrodopamine, roughness of the different PNDA films, height profiles of scratched films before and in nonirradiated areas, camera images of the patterned and nonirradiated films, XP survey spectra and high-resolution C 1s, N 1s, and O 1s XP spectra of the different films before and after irradiation and inside and outside the patterning and elemental composition of those films (PDF)
