# Supplementary material for: Lighting up Polynitrodopamine: from Electropolymerization to Photopatterning of Ultrathin Films
Source: ACS Appl Mater Interfaces. 2026 Apr 8;18(15):22274–86. doi: 10.1021/acsami.6c00129 (PMC13107374; doi:10.1021/acsami.6c00129)
Supplement: Supplementary file 1 [file am6c00129_si_001.pdf]

Supporting Information

for

Lighting Up Polynitrodopamine: From Electropolymerization to Photopatterning of Ultrathin Films

Marcel Boecker[a], Julia Moser[a], Tommaso Marchesi D'Alvise[a], Christof Neumann[b], Sean Harvey[a], Andrey Turchanin[b,c], Christopher V. Synatschke[a *], Tanja Weil[a *]

a Department for Synthesis of Macromolecules, Max Planck Institute for Polymer Research, 55128 Mainz, Germany

b Institute of Physical Chemistry, Friedrich Schiller University Jena, 07743 Jena, Germany

c Abbe Center of Photonics (ACP), 07745 Jena, Germany

Corresponding Author

Christoper V. Synatschke – Department for Synthesis of Macromolecules, Max Planck Institute for Polymer Research, 55128 Mainz, Germany; Email: synatschke@mpip-mainz.mpg.de

Tanja Weil – Department for Synthesis of Macromolecules, Max Planck Institute for Polymer Research, 55128 Mainz, Germany; Email: weil@mpip-mainz.mpg.de

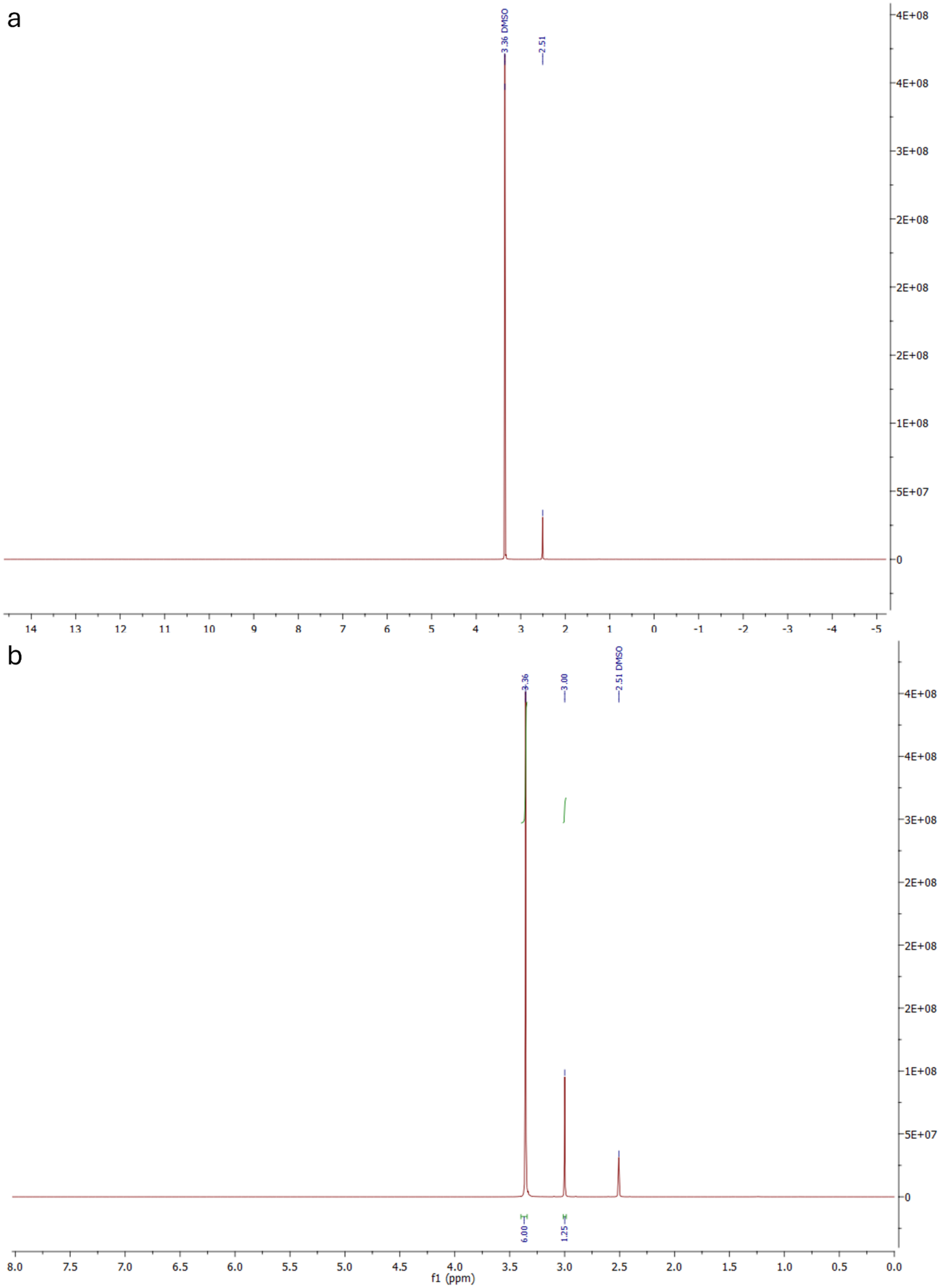
a
3.36 DMSO
2.51
4E+08
4E+08
3E+08
2E+08
2E+08
2E+08
1E+08
5E+07
0
14 13 12 11 10 9 8 7 6 5 4 3 2 1 0 -1 -2 -3 -4 -5
b
3.36
3.00
2.51 DMSO
4E+08
4E+08
3E+08
2E+08
2E+08
2E+08
1E+08
5E+07
0
6.00
1.25
8.0 7.5 7.0 6.5 6.0 5.5 5.0 4.5 4.0 3.5 3.0 2.5 2.0 1.5 1.0 0.5 0.0
f1 (ppm)

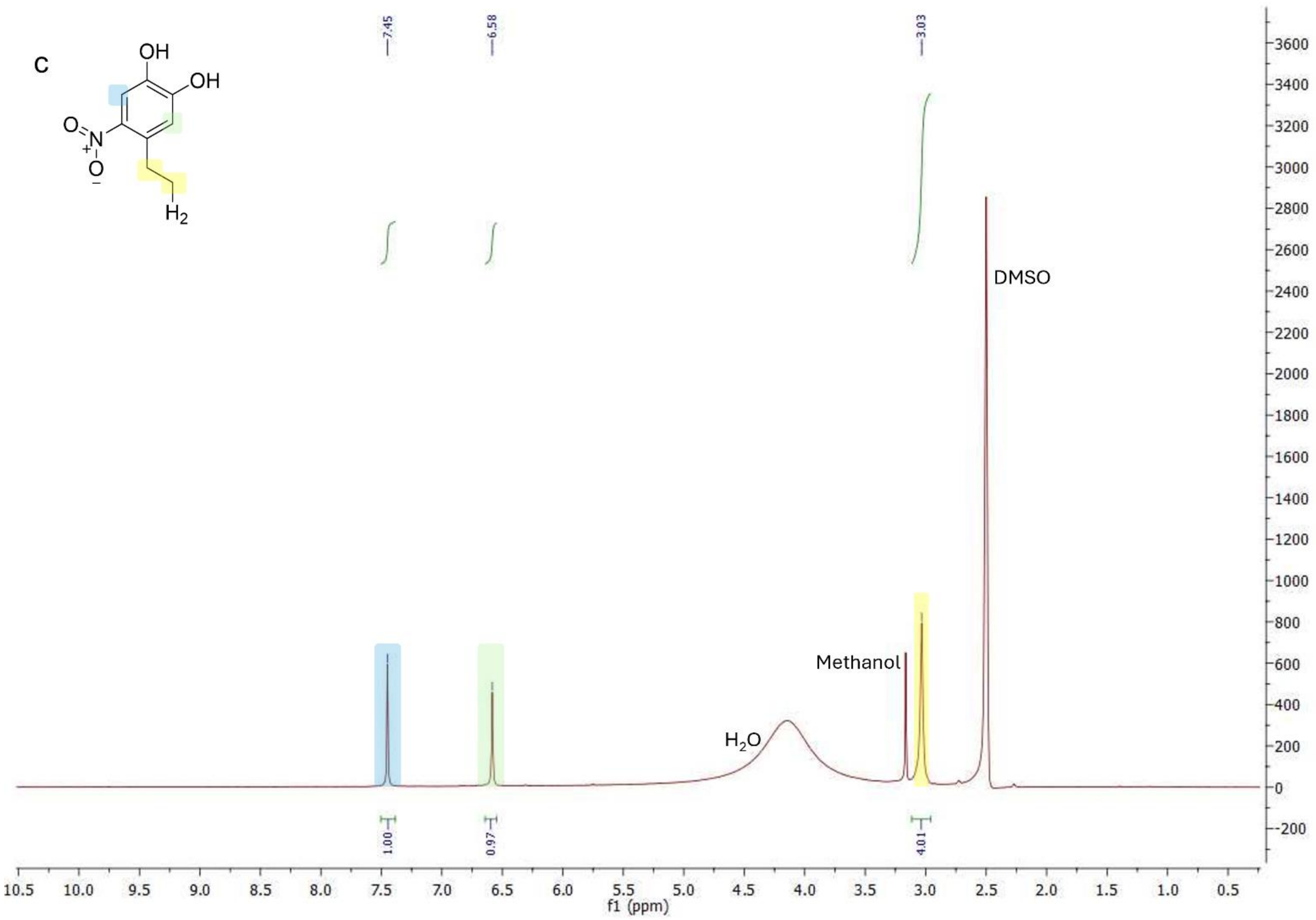

Figure S 1: (a) ^{1}H-NMR spectrum of pure DMSO-d_6 showing only the residual DMSO-d_5H peak (2.51 ppm) and a sharp water peak (3.36 ppm) originating from trace water content in the solvent. (b) ^{1}H-NMR spectrum of the standard dimethyl sulfone (DMS) in DMSO-d_6, displaying the DMSO-d_5H (2.51 ppm) and water (3.36 ppm) peaks, along with the characteristic DMS signal (3.00 ppm). (c) ^{1}H-NMR Spectrum of nitrodopamine in DMSO-d_6, showing all expected nitrodopamine signals. The water peak (3.59 ppm) is significantly broadened and downfield-shifted, attributed to proton exchange and hydrogen bonding interactions between nitrodopamine and residual water in the solvent.

Table S 1: Surface roughness of the different PNDA films, with the reported values of PDA in literature[1].

	Cycles	Roughness (nm)
PNDA	5	1.54
	10	1.83
	15	2.61
PDA	5	1.725
	10	1.453
	15	2.2

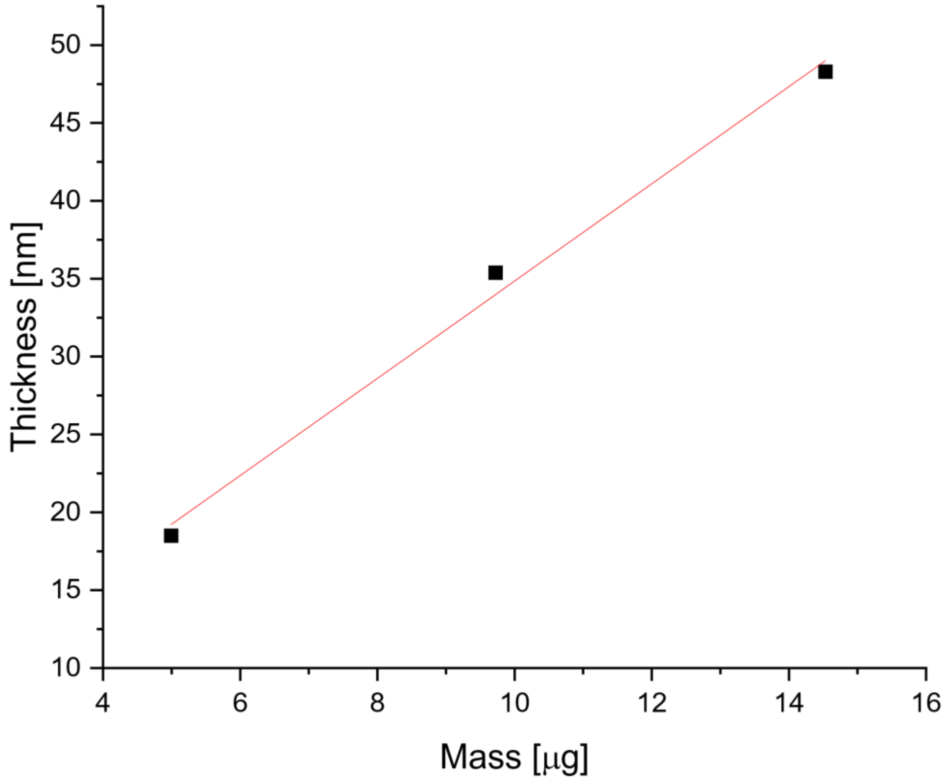

Figure S 2: Correlation between deposited mass (from eQCM) and film thickness (from AFM) of a PNDA film after 5, 10, and 15 cycles of electropolymerization. A linear fit (shown in red) was added to guide the eye, highlighting the near-linear relationship and indicating homogeneous film growth with increasing polymer deposition.

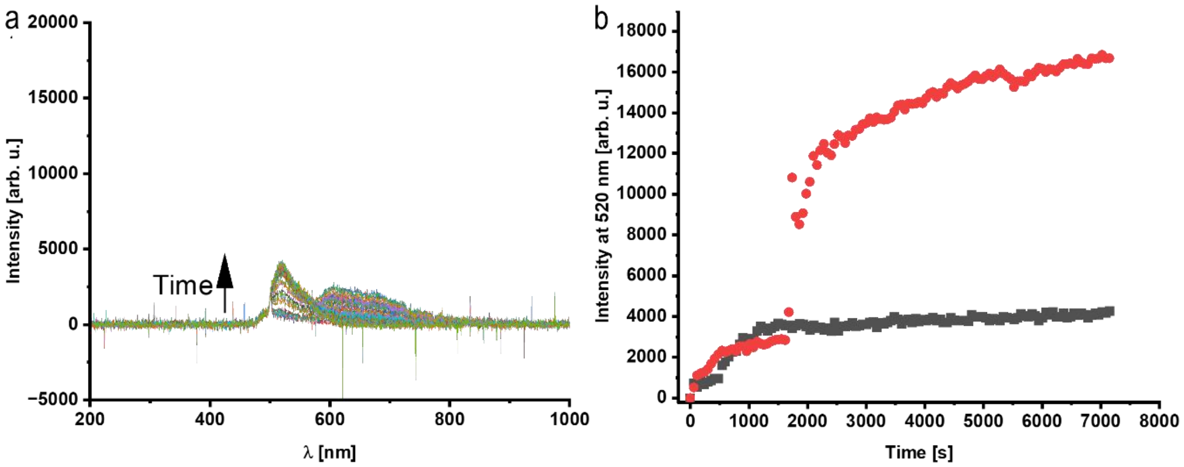

Figure S 3: (a) The recorded fluorescence spectra were measured over a duration of 2 hours during the irradiation of an FITC-functionalized PDA film with a 365 nm LED. (b) The intensity of the FITC at 520 nm in solution over the 2 hour irradiation process for PDA (black) and PNDA (red).

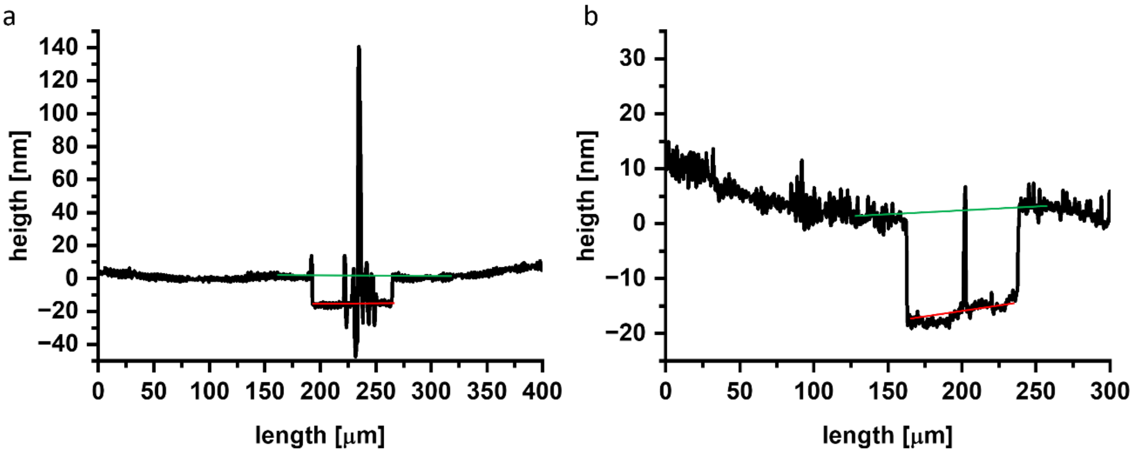

Figure S 4: Height profiles of a 10-cycle PNDA film, irradiated in dry conditions, including the profile of the covered area of the film (A) and the scratch in the irradiated area (B), both with the fits for the height determination (in green the fit of the film, in red the bottom of the scratch).

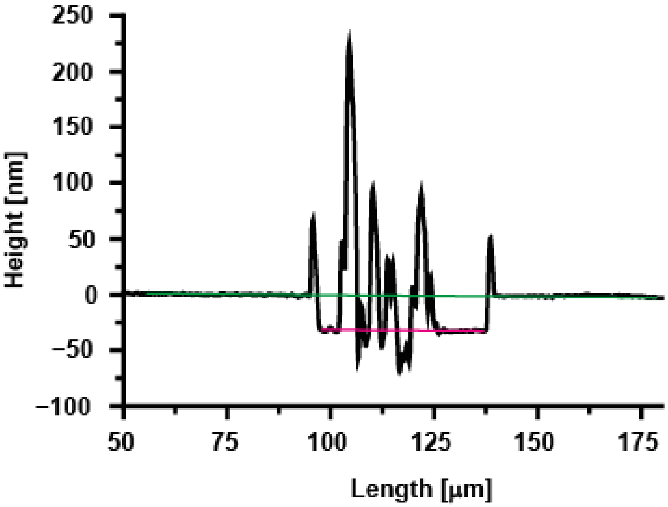

Figure S 5: Height profile measured by the profilometer of a mechanically-made scratch in a non-irradiated area with the fits for the height determination (in green the fit of the film, in red the bottom of the scratch).

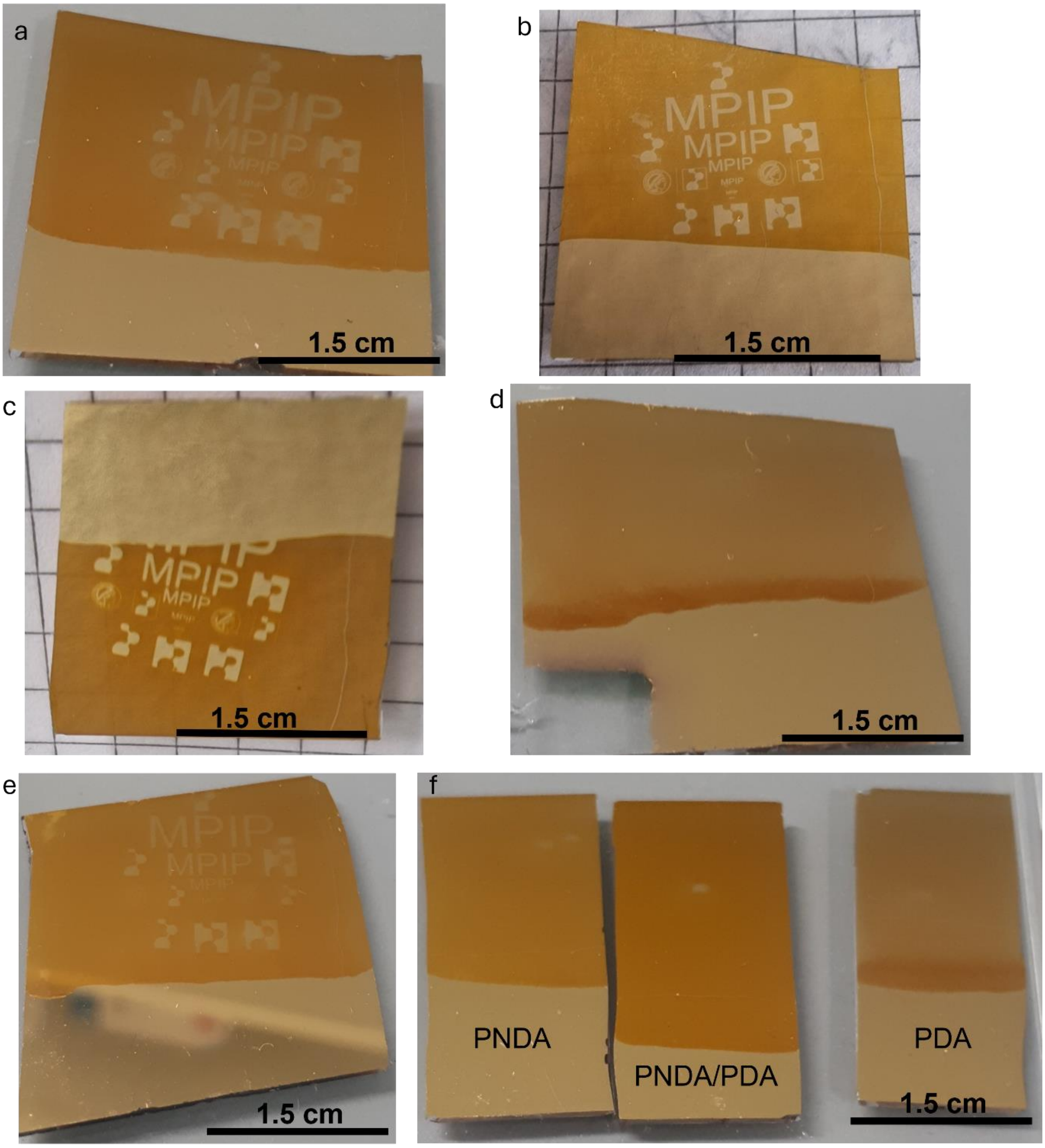

Figure S 6: (a) Camera image of the photopatterned 10 cycle PNDA film, (b) photopatterned 5 cycle PNDA film, (c) a photopatterned 15 cycle PNDA film, (d) a PDA film after irradiation, (e) a photopatterned PNDA/DA film and (f) non-irradiated reference films.

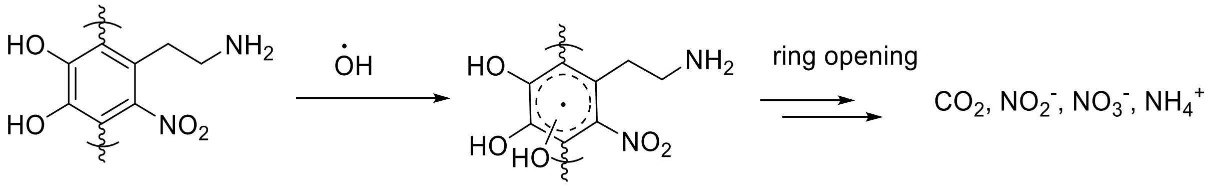

Figure S 7: Proposed degradation mechanism of PNDA, adapted from ref[2].

Table S 2: Degradation kinetics of 10 cycle PNDA films under UV irradiation, resulting in a degradation rate of 0.0131 min^{-1}.

Irradiation time [min]	Remaining film thickness [%]
0	100
30	76
60	36
120	~0

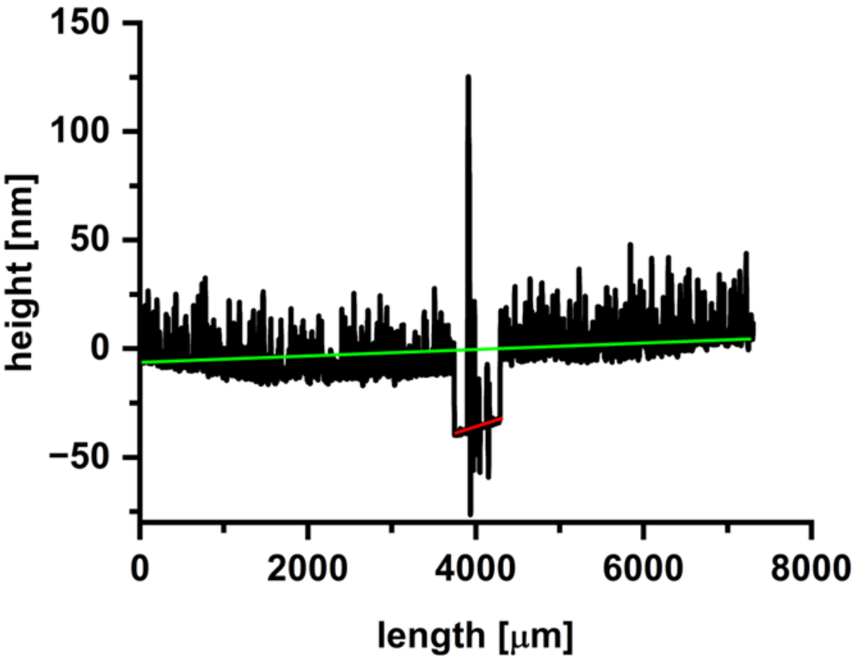

Figure S 8: Height profile of a scratched 10 cycle PNDA/PDA film with the fits for the height determination (in green the fit of the film, in red the bottom of the scratch).

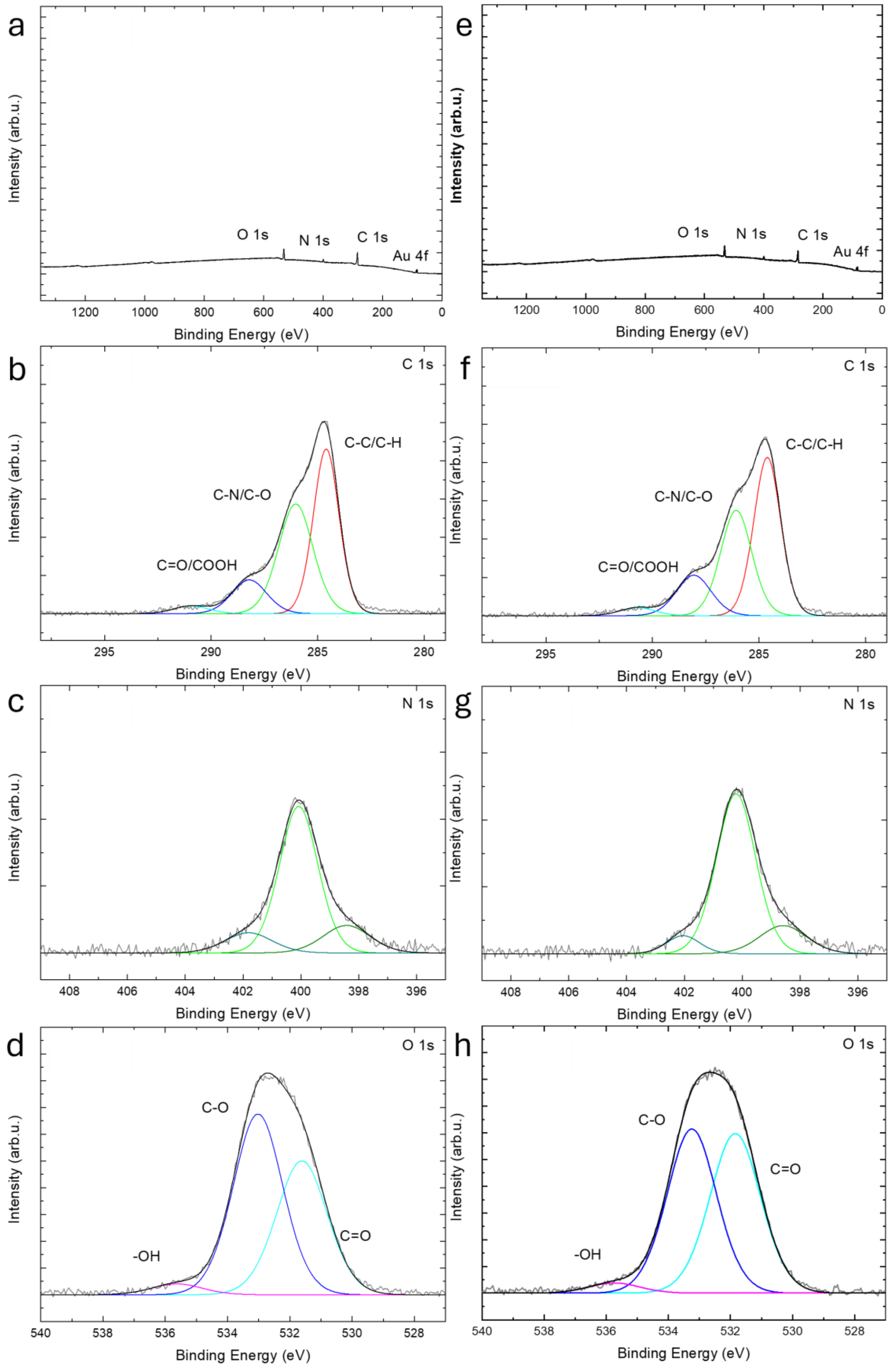

Figure S 9: XP survey spectrum and high-resolution XP spectra of polydopamine (PDA)with and without light exposure. (a) XP survey spectrum of as-prepared PDA (without light exposure). (b) high-resolution C 1s XP spectrum (no light exposure). (c) high-resolution N 1s XP spectrum (no light exposure). (d) high-resolution O 1s XP spectrum (no light exposure). (e) XP survey spectrum of PDA after irradiation. (f) high-resolution C 1s XP spectrum (after irradiation). (g) high-resolution N 1s XP spectrum (after irradiation). (h) high-resolution O 1s XP spectrum (after irradiation).

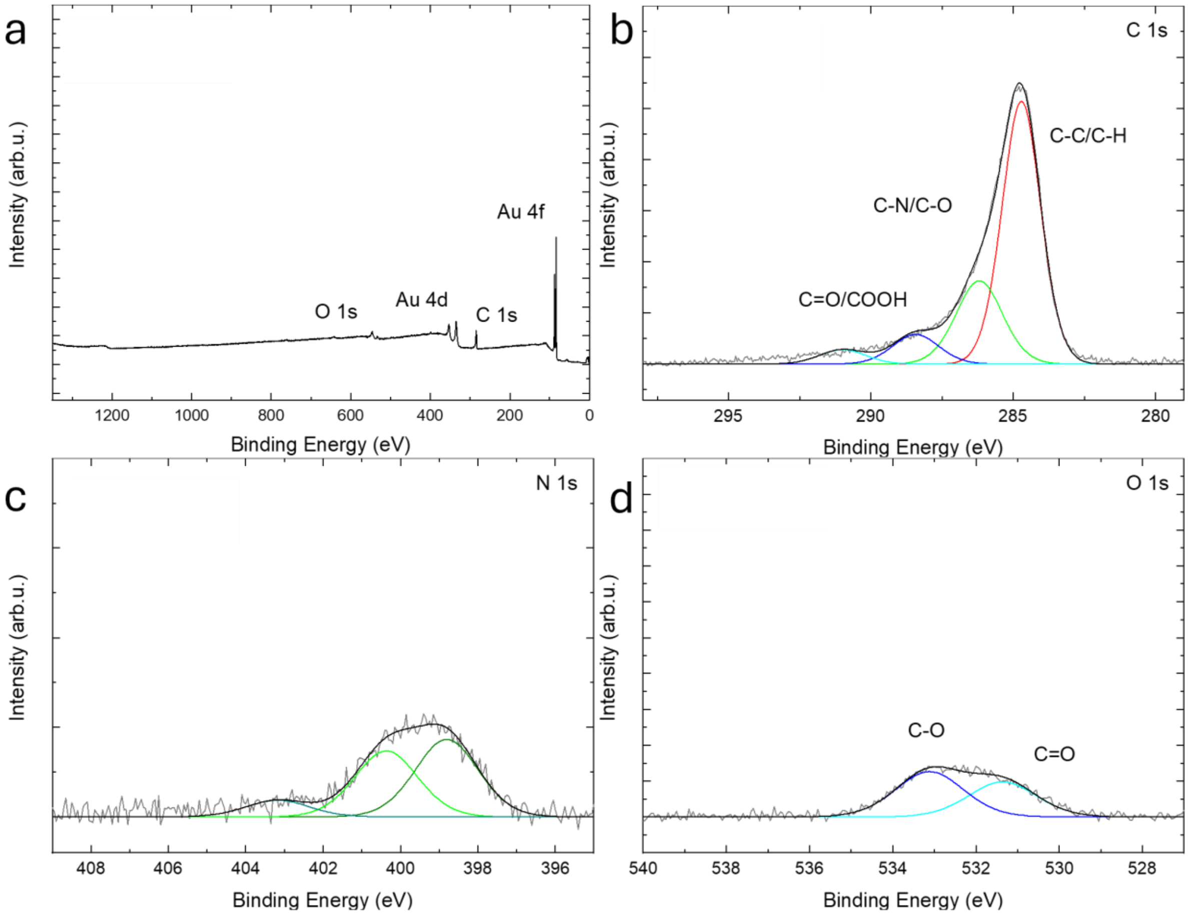

Figure S 10: XP survey spectrum and high-resolution XP spectra of polydopamine (PDA) after 15 seconds of Ar^+ sputtering. (a) XP survey spectrum. (b) high-resolution C 1s XP spectrum. (c) high-resolution N 1s XP spectrum. (d) high-resolution O 1s XP spectrum.

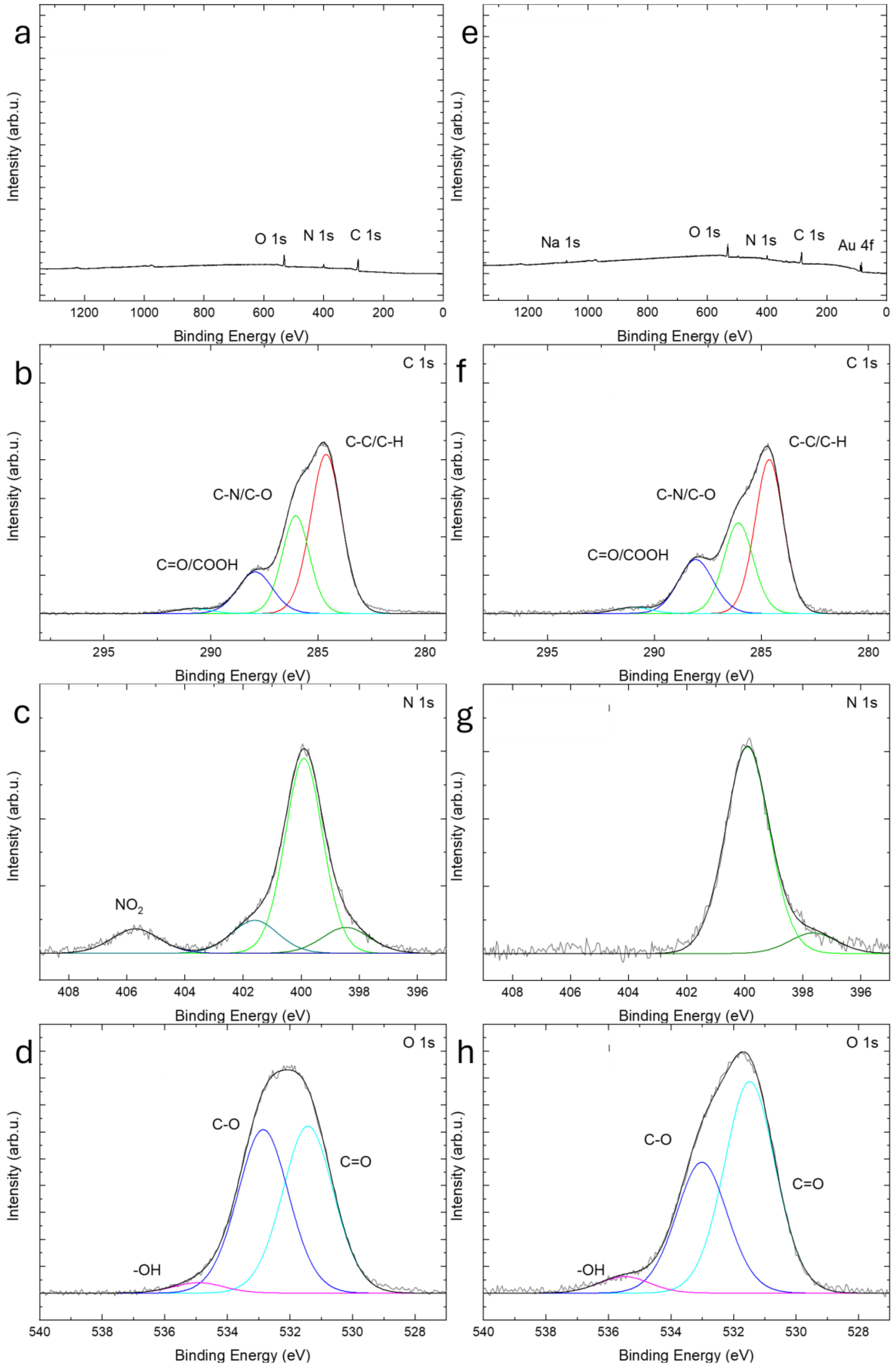

Figure S 11: XP survey spectrum and high-resolution XP spectra of polynitrodopamine (PNDA) with and without light exposure. (a) XP survey spectrum of as-prepared PNDA (without light exposure). (b) high-resolution C 1s XP spectrum (no light exposure). (c) high-resolution N 1s XP spectrum (no light exposure). (d) high-resolution O 1s XP spectrum (no light exposure). (e) XP survey spectrum of PNDA after irradiation. (f) high-resolution C 1s XP spectrum (after irradiation). (g) high-resolution N 1s XP spectrum (after irradiation). (h) high-resolution O 1s XP spectrum (after irradiation).

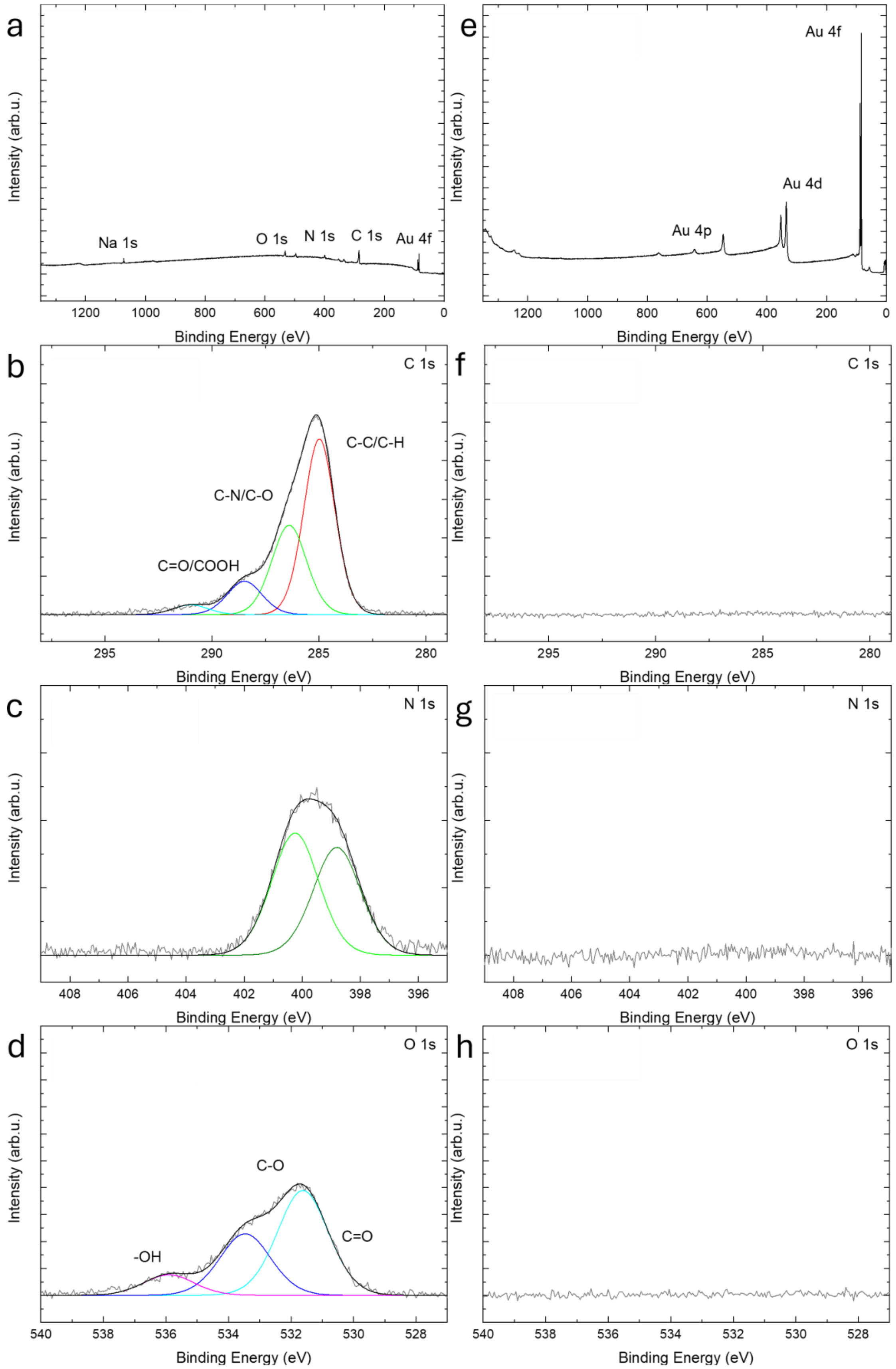

Figure S 12: XP survey spectrum and high-resolution XP spectra of PNDA in non-patterned and patterned sputtered areas. (a) XP survey spectrum of PNDA in the non-patterned area after 15 seconds of sputtering. (b) high-resolution C 1s XP spectrum (non-patterned area after sputtering). (c) high-resolution N 1s XP spectrum (non-patterned area after sputtering). (d) high-resolution O 1s XP spectrum (non-patterned area after sputtering). (e) XP survey spectrum of PNDA after 15 seconds of sputtering inside the pattern. (f) high-resolution C 1s XP spectrum (patterned area after sputtering). (g) high-resolution N 1s XP spectrum (patterned area after sputtering). (h) high-resolution O 1s XP spectrum (patterned area after sputtering).

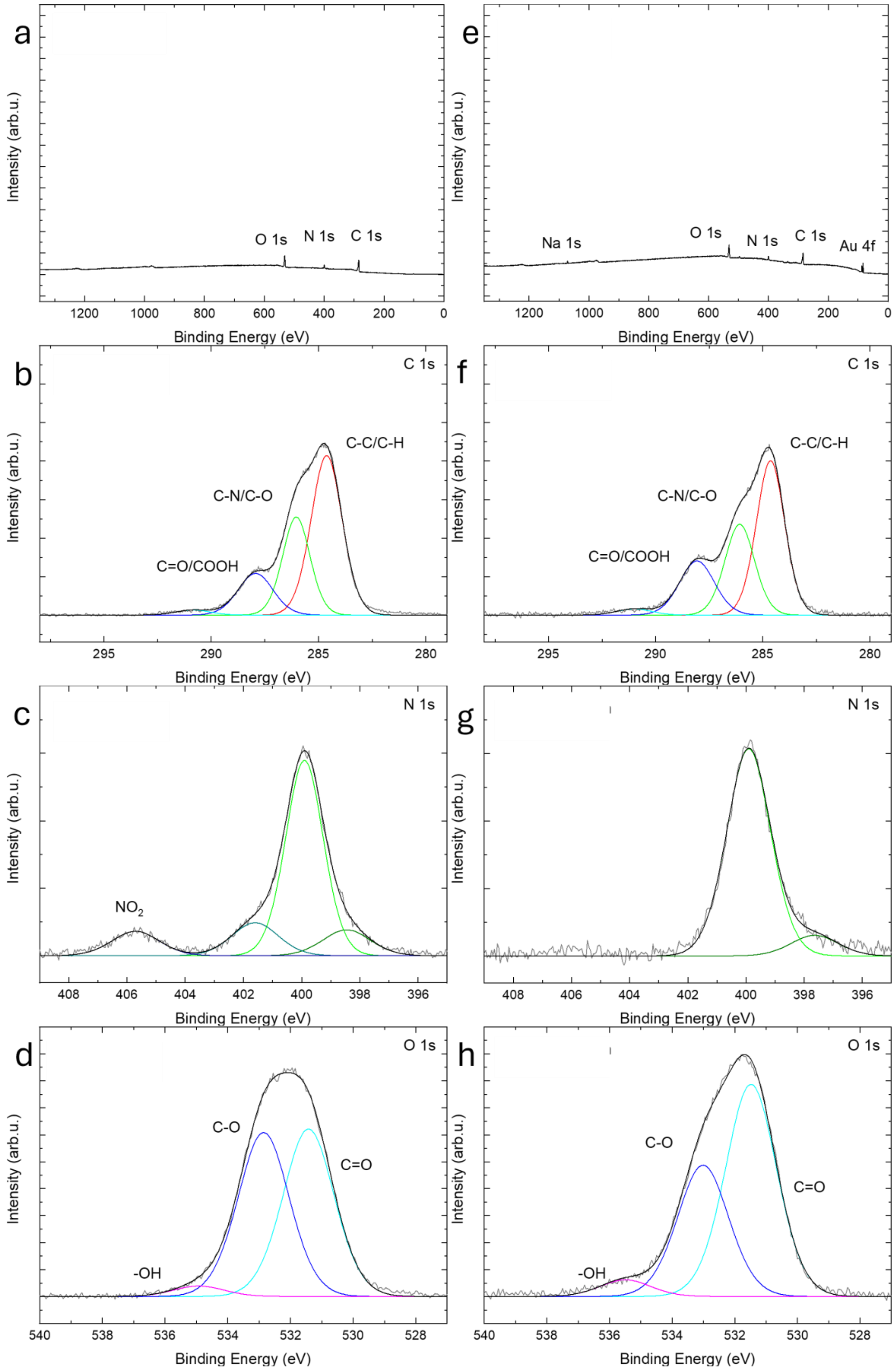

Figure S 13: XP survey spectrum and high-resolution XP spectra of the copolymer polynitrodopamine/polydopamine (PNDA/PDA) with and without light exposure. (a) XP survey spectrum of as-prepared PNDA/PDA (without light exposure). (b) high-resolution C 1s XP spectrum (no light exposure). (c) high-resolution N 1s XP spectrum (no light exposure). (d) high-resolution O 1s XP spectrum (no light exposure). (e) XP survey spectrum of PNDA/PDA after irradiation. (f) high-resolution C 1s XP spectrum (after irradiation). (g) high-resolution N 1s XP spectrum (after irradiation). (h) high-resolution O 1s XP spectrum (after irradiation).

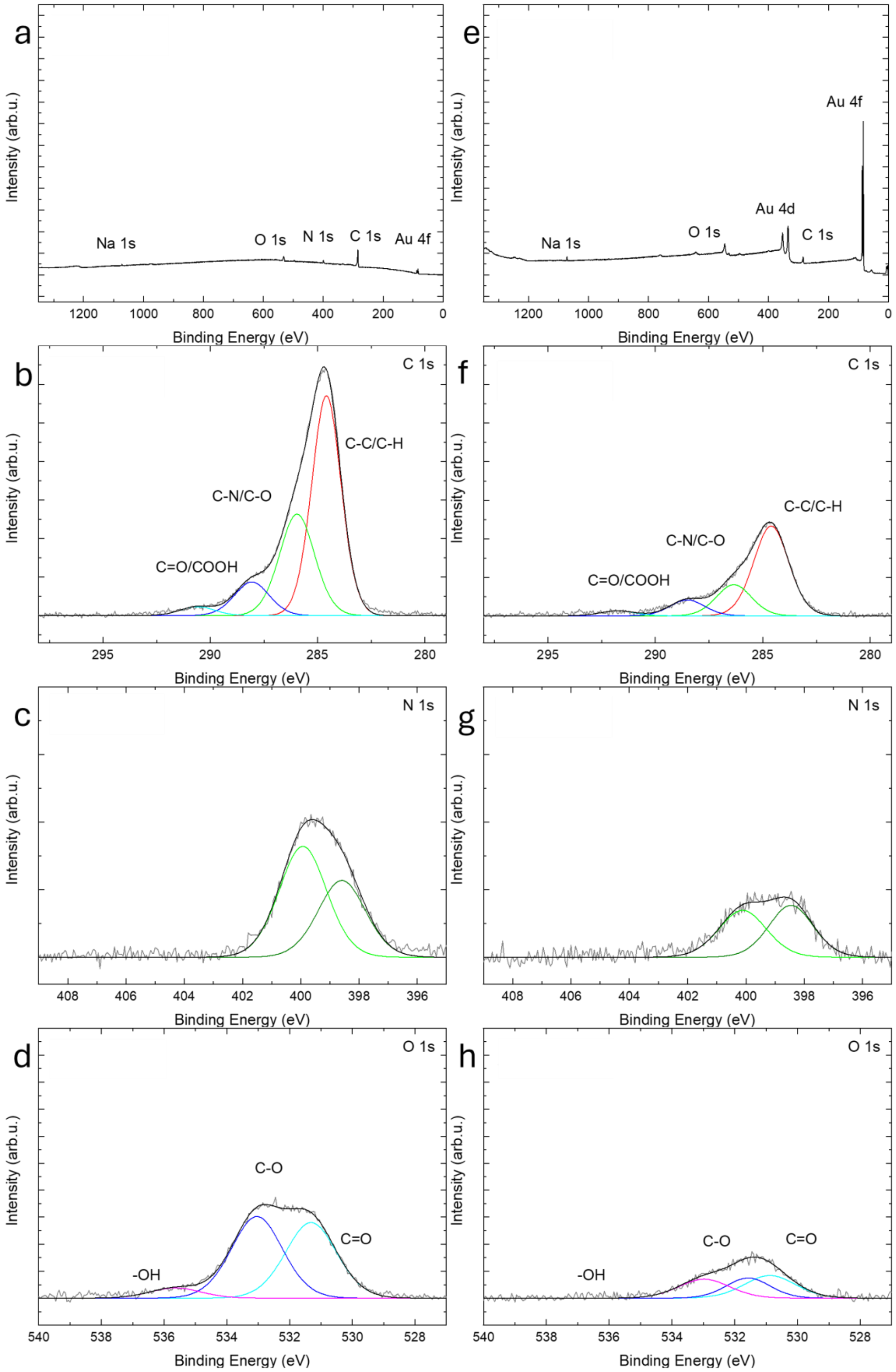

Figure S 14: XP survey spectrum and high-resolution XP spectra of PNDA/PDA in non-patterned and patterned sputtered areas. (a) XP survey spectrum of PNDA/PDA in the non-patterned area after 15 seconds of sputtering. (b) high-resolution C 1s XP spectrum (non-patterned area after sputtering). (c) high-resolution N 1s XP spectrum (non-patterned area after sputtering). (d) high-resolution O 1s XP spectrum (non-patterned area after sputtering). (e) XP survey spectrum of PNDA/PDA after 15 seconds of sputtering inside the pattern. (f) high-resolution C 1s XP spectrum (patterned area after sputtering). (g) high-resolution N 1s XP spectrum (patterned area after sputtering). (h) high-resolution O 1s XP spectrum (patterned area after sputtering).

Table S 3: Elemental composition (unit at%) of the PDA, PNDA, and PNDA/DA determined by XPS of the untreated films (as-prepared), Ar^+-sputtered regions of the untreated film (sputtered), irradiated regions (irradiated/pattern), and after Ar^+ sputtering inside the patterned regions (pattern+sputtered).

Elements	C	N	O	Au	Na	Si
PDA	66.5	7.5	23.2	0.6	0.1	2.1
PDA sputtered	77.3	6.4	7.5	8.8	-	-
PDA irradiated	65.0	7.9	23.9	0.6	0.4	2.2
PNDA	60.5	11.2	25.8	0.1	1.3	1.1
PNDA sputtered	70.4	10.3	13.5	2.8	3.0	-
PNDA pattern	41.6	6.7	18.3	29.9	3.5	-
PNDA pattern+sputtered	-	-	-	100	-	-
PNDA/DA	63.3	10.4	24.1	0.1	0.6	1.5
PNDA/DA sputtered	78.1	8.2	11.8	0.9	1.0	-
PNDA/DA pattern	61.3	9.1	24.8	1.5	1.5	1.8
PNDA/DA pattern+sputtered	50.0	6.2	6.1	33.6	4.1	-

References:

(1) Marchesi D'Alvise, T.; Sunder, S.; Hasler, R.; Moser, J.; Knoll, W.; Synatschke, C. V.; Harvey, S.; Weil, T. Preparation of Ultrathin and Degradable Polymeric Films by Electropolymerization of 3-Amino- L-tyrosine. *Macromol Rapid Commun* **2023**, *44* (16). https://doi.org/10.1002/marc.202200332.

(2) Maurino, V.; Minero, C.; Pelizzetti ~', E.; Piccinini ~, P.; Serpone, N.; Hidaka, H. The Fate of Organic Nitrogen under Photocatalytic Conditions: Degradation of Nitrophenols and Aminophenols on Irradiated TiO_2. *J. Photochem. Photobiol. A-Chem* **1997**, 109, 171-176. https://doi.org/10.1016/S1010-6030(97)00124-X
